# Experiment-Based Optimization of LED Spectral Irradiance Ratios for Enhancing Biomass, Secondary Metabolite, and Essential Oil Yields and Compositions of *Ocimum* × *africanum* Lour. Under Controlled Environmental Conditions

**DOI:** 10.3390/molecules31152618

**Published:** 2026-07-27

**Authors:** Ha Thi Thu Chu, Thi Nghiem Vu, Quang Cong Tong, Tran Quoc Tien, Thanh Phuong Nguyen, Thuy Thi Thu Dinh, Isabell Pappert, Felix Wirth, Luca Jokic, Alexander Schiesser, Khanh Quoc Tran

**Affiliations:** 1 Institute of Biology, Vietnam Academy of Science and Technology (VAST), 18 Hoang Quoc Viet, Nghia Do, Ha Noi 10000, Vietnam; 2 Faculty of Natural Sciences, Electric Power University, 235 Hoang Quoc Viet, Nghia Do, Ha Noi 10000, Vietnam; nghiemvt78@gmail.com; 3 Institute of Materials Science, Vietnam Academy of Science and Technology, 18 Hoang Quoc Viet, Nghia Do, Ha Noi 10000, Vietnam; congtq@ims.vast.ac.vn (Q.C.T.); tientq@ims.vast.ac.vn (T.Q.T.); 4 Faculty of Engineering Physics, Hanoi University of Science and Technology, No.1 Dai Co Viet, Bach Mai, Ha Noi 10000, Vietnam; phuong.nguyenthanh@hust.edu.vn; 5 Institute of Chemistry, Vietnam Academy of Science and Technology, 18 Hoang Quoc Viet, Nghia Do, Ha Noi 10000, Vietnam; dttthuy@ich.vast.vn; 6 Laboratory of Adaptive Lighting Systems and Visual Processing, Technical University of Darmstadt, Hochschulstr. 4a, 64289 Darmstadt, Germany; pappert@lichttechnik.tu-darmstadt.de (I.P.); wirth@lichttechnik.tu-darmstadt.de (F.W.); 7 Department of Applied Plant Sciences, Faculty of Biology, Technical University of Darmstadt, Schnittspahnstr. 10, 64287 Darmstadt, Germany; luca.jokic@t-online.de; 8 Department of Chemistry, Mass Spectrometry, Technical University of Darmstadt, Peter-Grünberg-Straße 4, 64287 Darmstadt, Germany; alexander.schiesser@tu-darmstadt.de

**Keywords:** essential oil composition, geranial, LED light spectra, lemon basil, neral, *Ocimum basilicum* var. *pilosum*, secondary metabolite accumulation, spectral irradiance ratios

## Abstract

This study evaluated the effects of different LED spectral irradiance ratios on the growth, secondary metabolites, and essential oil characteristics of *Ocimum* × *africanum* Lour. cultivated for four weeks under controlled conditions. Four LED lighting treatments with different red, blue, green, ultraviolet-A, and far-red light ratios, and a treatment in greenhouse as low-light control were used. The constant 16 h photoperiod and light intensity of 220 µmol·m^−2^·s^−1^ were maintained. The F2 treatment (UV-A:B:R:Fr = 6.60:45.15:29.23:19.02) promoted the greatest plant height (76.62 cm), and chlorophyll a (7.41 mg/100 g, FW), chlorophyll b (4.73 mg/100 g, FW), and total phenolic (25.78 mg/g, FW) concentrations. F1 treatment (B:G:R:Fr = 17.14:29.8:47.4:5.66) produced significantly higher (*p* < 0.01) biomass (8.3 ton/ha, FW), oil yield (10.89 L/ha), and carotenoid (3.74 mg/100 g, FW) than the others. Essential oils contained 12–15 compounds, dominated by neral (27.5–37.3%), geranial (41.1–49.9%), and (*E*)-*β*-caryophyllene (2.4–9.9%), while the highest contents of oil (0.83%, DW), anthocyanin (16.50 mg/100 g, FW), and total flavonoid (14.17 mg/g, FW) were obtained under F4 (B:G:R:Fr = 13.85:43.50:39.30:3.35). These findings demonstrate that optimized LED spectra can effectively improve both productivity and phytochemical quality in *O. africanum* through regulating both primary and secondary metabolism.

## 1. Introduction

Lemon basil (*Ocimum* × *africanum* Lour.), belonging to the Lamiaceae family, is an aromatic annual herb widely distributed in tropical and subtropical Asia, particularly in Vietnam and China. This plant is known by the synonym “*Ocimum basilicum* var. *pilosum* (Willd.) Benth.)” in Vietnamese literature. It grows 30–80 cm tall with quadrangular, branched stems and has leaves that are ovate to broadly lanceolate, with slightly serrated margins and light green laminae. The plant emits a distinctive fragrance attributed to its abundant essential oil glands and high essential oil content. Adapted to warm, humid, and high-light environments, lemon basil grows rapidly and accumulates substantial biomass, making it suitable for commercial production of essential oils and bioactive phytochemicals [1]. Traditionally, its seeds are used in refreshing beverages, while the plant has been employed in folk medicine for treating snake bites and skin inflammation [2]. The leaves and flowers are rich in fragrant essential oils, making lemon basil an important subject of research for applications in the food, pharmaceutical, and cosmetic industries [3].

Essential oil accumulation occurs primarily in the leaves and flowers of lemon basil, with minimal content in the stem. During flowering, oil content can reach up to 1.25% in flowers and approximately 1.14% in leaves (dry weight). Previous studies showed that citral is the dominant compound, accounting for 39.73–40.64% of flower oil and 50.22–54.81% of leaf oil [3]. However, the chemical composition of lemon basil essential oil varies markedly with geographic origin and environmental conditions. Major constituents reported include linalool (0.6–46.1%), and methyl chavicol (3.7–84.0%), citral (16.6–33.6%) in lemon basil (*syn. Ocimum* × *citriodorum*) in the United States [4], linalool (29.68%), and (*Z*)-cinnamic acid methyl ester (21.49%) in China [1]. However, the RI values of the constituent compounds of the essential oil in lemon basil were not published in these two studies, leading to low reliability of these results. Other studies reported the chemotype “citral” of lemon basil, such as a diverse combinations of neral (21.1–36.8%), geranial (15.6–33.4%), linalool, and methyl chavicol across different regions of Thailand [5], and neral (15.6%), geranial (22.8%), β-caryophyllene (11.7%), and α-humulene (12.6%) in basil of Indonesia [6]. A recent investigation revealed substantial geographical variation in the chemical composition of lemon basil essential oil across different regions of China. Specifically, the contents of estragole (18.4%, 20.4%, and 21.4%), β-caryophyllene (13.3%, 7.9%, and 13.9%), and α-caryophyllene (11.1%, 7.8%, and 11.2%) were identified in essential oils obtained from Henan, Shandong, and Anhui provinces, respectively [7]. Besides geographical factors, other environmental conditions also affect the phytochemical characteristics of lemon basil, such as nutrient media supplemented with natural compound additives [8], the growth stages of a plant, which include the pre-flowering stage, the full flowering stage, and the post-flowering stage [9], and different organic fertilization conditions [10].

Lemon basil is widely utilized in the culinary, fragrance, and pharmaceutical industries, primarily through the extraction of its essential oil, particularly during the full-flowering stage [11]. In addition to its aromatic properties, lemon basil essential oil exhibits notable biological activities, particularly strong antifungal effects against plant-pathogenic fungi such as *Fulvia fulva* and *Fusarium solani* var. *coeruleum*, with EC_50_ values below 20 ppm [1]. The antioxidant activity of lemon basil essential oil was shown to be stronger than that of the extract, and there are differences between parts of the plant. Specifically, the essential oil from the leaves has the strongest antioxidant activity, followed by the flowers and the stem [12]. Essential oil from lemon basil in Indonesia was studied for its potential to inhibit bacterial growth on tofu and chicken fillets during storage and extend their shelf life up to, respectively, 4 and 6 days [6,13]. In general, many species in the genus *Ocimum* have biological activities, such as the ability to scavenge free radicals, reduce cellular oxidative stress, and support cardiovascular health. Studies on various *Ocimum* species showed that the total phenolic and total flavonoid content correlates strongly with the antioxidant activity of extracts and essential oils [14]. The phenolic compounds in lemon basil, in the form of rosmarinic and caffeic acids, are effective antioxidants, and it has also been shown that they can strongly inhibit some strains of harmful fungi [15,16]. As in all chlorophyll-containing green plants, chlorophyll in lemon basil plays a central role in capturing light energy for photosynthesis. Through this process, absorbed light energy is converted into chemical energy that drives carbon fixation and other metabolic activities [17]. Chlorophyll occurs in several forms, with chlorophyll *a* and chlorophyll *b* being the primary photosynthetic pigments. These pigments differ in their absorption characteristics and contribute complementary functions in light harvesting. Chlorophyll *a* predominantly absorbs R light around 665–680 nm, whereas chlorophyll *b* exhibits stronger absorption in the B region near 460 nm, thereby extending the spectral range available for photosynthesis [18]. In lemon basil, chlorophyll-related studies have primarily focused on chlorophyll fluorescence analysis as a non-destructive indicator of photosynthetic performance, plant growth, and stress responses under varying environmental conditions [19].

Under the increasing pressures of climate change, crop productivity and quality are becoming more susceptible to environmental fluctuations, thereby compromising the stability and sustainability of agricultural production systems. For medicinal plants, improving the consistency and quality of raw materials requires the optimization and standardization of cultivation, harvesting, and processing protocols. Environmental factors play a crucial role in regulating plant growth and secondary metabolite production, among which light is one of the most influential. In addition to serving as the primary energy source for photosynthesis, light acts as an essential environmental signal that regulates plant growth, development, and physiological processes through photoreceptor-mediated pathways [20,21,22]. Light quality has been shown to influence plant morphology, photosynthetic efficiency, organ development, and the biosynthesis of numerous primary and secondary metabolites, including proteins, carbohydrates, vitamins, phenolic compounds, flavonoids, and anthocyanins [20]. Among the light spectra, red (R) and blue (B) lights are considered the most effective in driving photosynthesis, as chlorophyll pigments predominantly absorb radiation within these regions. Nevertheless, other spectral components, including ultraviolet-A (UV-A), green (G), and far-red (Fr) light, also serve as important regulatory signals influencing plant physiology and development [23]. In particular, UV-A radiation has attracted considerable attention because of its ability to enhance photosynthetic performance and stimulate the biosynthesis of bioactive secondary metabolites, including phenolics and flavonoids [24]. For example, the supplementation of UV-A radiation to a red–white light spectrum (20% R:80% W) during the final three days before harvest significantly increased shoot fresh weight, plant height, and the number of leaves and branches in coriander [25]. Similarly, exposure to UV-A radiation (385 nm, 30 W m^−2^) for five days increased both biomass yield and phenolic content in kale (*Brassica oleracea* var. *acephala*) [26]. In lettuce (*Lactuca sativa*), UV-A supplementation (365 nm, 10–30 μmol·m^−2^·s^−1^) for 13 days promoted fresh and dry biomass accumulation, leaf expansion, and antioxidant activity compared with plants grown without UV-A radiation [27]. Likewise, the combination of R light (215 μmol·m^−2^·s^−1^) and UV-A light (35 μmol·m^−2^·s^−1^) significantly enhanced flavonoid accumulation in tomato (*Solanum lycopersicum*) relative to R light alone [28]. Although G light has traditionally been considered less efficient for photosynthesis, its deeper penetration into leaf tissues facilitates improved carbon assimilation and can enhance crop productivity [29]. Beyond its role in light absorption, G light also regulates key physiological processes, including stomatal behavior, canopy architecture, and resource-use efficiency, thereby contributing to plant performance under controlled-environment conditions [30].

Fr radiation, which is only weakly absorbed by chlorophyll and is largely transmitted or reflected by leaves [17], has also been shown to substantially affect plant growth and productivity. Zhen & van Iersel [31] found that Fr light increased net photosynthesis in lettuce under R-B lighting by improving light-use efficiency in photosynthetic reactions. Likewise, supplementing a R–B–W spectrum (70:20:10) with Fr radiation significantly increased plant height, internode length, leaf area, and fresh biomass in coriander [32]. In another study, the addition of 6% Fr light to a R–B spectrum increased total phenolic content and ascorbic acid concentration compared with R–B lighting alone [33]. Similar growth-promoting effects have been reported in ornamental crops, where the addition of 16–64 μmol·m^−2^·s^−1^ Fr light to a R–B background increased shoot dry weight by 28–50% in geranium and snapdragon [34]. Furthermore, Zou et al. [35] observed increases of 49% in leaf area and 39% in biomass production in lettuce when 50 μmol·m^−2^·s^−1^ Fr light was added to a R–B lighting regime. Collectively, these findings suggest that Fr supplementation is an effective strategy for enhancing light interception, photosynthetic performance, and biomass accumulation.

Increasing evidence suggests that light spectral composition strongly influences essential oil synthesis and other specialized metabolites in aromatic plants. McAusland et al. [36] demonstrated that in coriander (*Coriandrum sativum*), mixed R:B (1:1) and R:B:G (35.8:37.8:26.4) spectra not only affected growth but also increased major essential oil compounds by three- to four-fold compared with monochromatic R or B light. This enhanced aromatic profile was linked to stimulated secondary metabolite biosynthesis involved in plant defense and ecological adaptation.

For *Ocimum* × *africanum*, to date, some efforts have been devoted to understanding how light intensity and spectral ratios affect plant growth and development. Mat Daud et al. [37] evaluated the effects of four light intensities (50, 80, 120, and 150 µmol·m^−2^·s^−1^) under a constant R-to-B ratio of 4:1 and observed significant variations in leaf, stem, and root biomass, as well as photosynthetic performance, in lemon basil. Net photosynthetic rate increased with increasing light intensity and was highest under 150 µmol·m^−2^·s^−1^. Likewise, the highest fresh and dry biomass were recorded under the 120 and 150 µmol·m^−2^·s^−1^ treatments. These findings suggest that light intensity is a key determinant of photosynthetic efficiency and biomass production in lemon basil when spectral composition is held constant. Daud et al. [38] reported that lemon basil (syn. *Ocimum × citriodurum* Vis.) grown under combined R and B (4:1) LED lighting at 160 µmol·m^−2^·s^−1^ with a nutrient solution EC of 2.6 mS cm^−1^ exhibited significantly greater growth, yield, and phenolic and flavonoid accumulation than plants grown under lower irradiance. Another study evaluated the influence of LED irradiance on biomass yield, antioxidant production, and antioxidant capacity in microgreens of five traditional Thai vegetable species, including lemon basil. Among the tested light intensities, a photosynthetic photon flux density (PPFD) of 330 µmol·m^−2^·s^−1^ proved most effective in promoting dry matter accumulation, resulting in higher dry biomass, total phenolic and flavonoid content, and free radical scavenging than plants exposed to PPFD levels of 220 or 110 µmol·m^−2^·s^−1^ and those grown under conventional fluorescent lighting (45 µmol·m^−2^·s^−1^) [39]. On the other hand, Pitaloka et al. [40] evaluated the effect of three LED spectra combined with different planting media and nutrient concentrations on lemon basil growth. The best performance was achieved under 100% white LED lighting with a 700 ppm nutrient solution, as evidenced by increased plant height, leaf area, fresh biomass, and chlorophyll content. However, the lighting treatments differed in both spectral quality and intensity, making it difficult to separate their effects. Specifically, the 100% W treatment had the highest PPFD (95.92 µmol·m^−2^·s^−1^), compared with 100% B (44.26 µmol·m^−2^·s^−1^) and R:B:W (67%:20%:13%) (37.33 µmol·m^−2^·s^−1^). So, its superior growth may reflect combined effects of light quality and intensity. A recent study showed that in *Ocimum* species, including lemon basil, high DLI (22.2–23.3 mol·m^−2^·d^−1^) combined with a 16 h photoperiod induced the earliest flowering, highlighting the importance of both light intensity and day length in regulating reproductive development [41]. Another recent study investigated the influence of cultivation environments and lighting conditions on the growth and essential oil characteristics of lemon basil, comparing field, greenhouse (without supplemental light), and LED-illuminated city farming systems. The results showed the highest plant height in greenhouse plants and the lowest in field-grown plants, while essential oil composition varied across environments, indicating strong environmental effects on secondary metabolism. However, the lack of reported retention index (RI) values limited the reliability and reproducibility of compound identification [42].

Despite growing evidence highlighting the pivotal role of light quality in regulating plant growth, physiology, and secondary metabolism, most studies on LED effects in lemon basil have focused on light intensity rather than spectral composition. Thus, the impact of different multispectral light ratios at a constant PPFD on growth, secondary metabolite and essential oil biosynthesis remains unclear. To address this gap, this study examined the effects of multispectral LED lighting with varying proportions of UV-A, B, G, R, and Fr lights on lemon basil growth, secondary metabolite, and essential oil production under controlled conditions with similar PPFD across treatments, allowing the effects of the light spectrum to be isolated. Five lighting formulations were designed as follows:

- B:G:R:Fr = 17.14:29.8:47.4:5.66 (F1);
- UV-A:B:R:Fr = 6.60:45.15:29.23:19.02 (F2);
- UV-A:B:R:Fr = 3.30:47.37:29.24:20.09 (F3);
- B:G:R:Fr = 13.85:43.50:39.30:3.35 (F4);
- F5—consisting of natural sunlight transmitted through the greenhouse at approximately 25% of ambient solar irradiance, which served as a low-light control.

The design of the four lighting treatments (F1–F4) was based on a systematic approach aimed at disentangling the individual and interactive effects of key spectral regions within the photosynthetically active and adjacent radiation ranges. Specifically, the spectral compositions were structured to allow for controlled variation in UV-A, B, G, R, and Fr light fractions while maintaining comparable overall PPFD across treatments. This design enables the isolation of spectral quality effects independent of differences in light intensity, thereby enabling the effects of spectral quality to be evaluated independently of irradiance, an experimental approach that facilitates a more rigorous assessment of spectral responses. Treatment F1 was developed using a white LED background supplemented with R light (660 nm), whereas F2 and F3 were composed of monochromatic LEDs (UV-A (360 nm), B (460 nm), R (660 nm), and Fr (730 nm)). Treatment F4 was based on a broad-spectrum white LED source. The selected spectral combinations were based on previous findings indicating that R and B light maximize photosynthetic performance, UV-A promotes secondary metabolite biosynthesis, Fr light enhances photosynthetic efficiency and biomass production, and G light improves canopy light penetration and overall light-use efficiency. The spectral distributions of the five used lighting treatments are illustrated in Section 4.1 in this paper.

Accordingly, the present study aimed to evaluate the effects of different LED spectral compositions on the growth, physiological performance, and phytochemical characteristics of lemon basil cultivated under controlled-environment conditions. Specifically, plant height, biomass production, photosynthetic pigments (chlorophyll a, chlorophyll b, and carotenoids), anthocyanin content, total phenolic content (TPC), total flavonoid content (TFC), essential oil yield, and essential oil composition were investigated. By systematically modifying the spectral distribution of LED lighting, this study sought to identify optimal light combinations that simultaneously maximize biomass productivity and the accumulation of high-value bioactive compounds. To the best of our knowledge, this is the first study to comprehensively assess the effects of multispectral LED lighting on growth, physiological traits, phytochemical accumulation, and essential oil composition in lemon basil. The findings are expected to provide new insights into light-mediated metabolic regulation and support the development of sustainable, precision-lighting strategies for the commercial production of high-quality aromatic and medicinal plants.

## 2. Results

### 2.1. The Effect of LED Light Conditions on the Biomass and Essential Oil Yield of Ocimum × africanum

**Figure 1:**
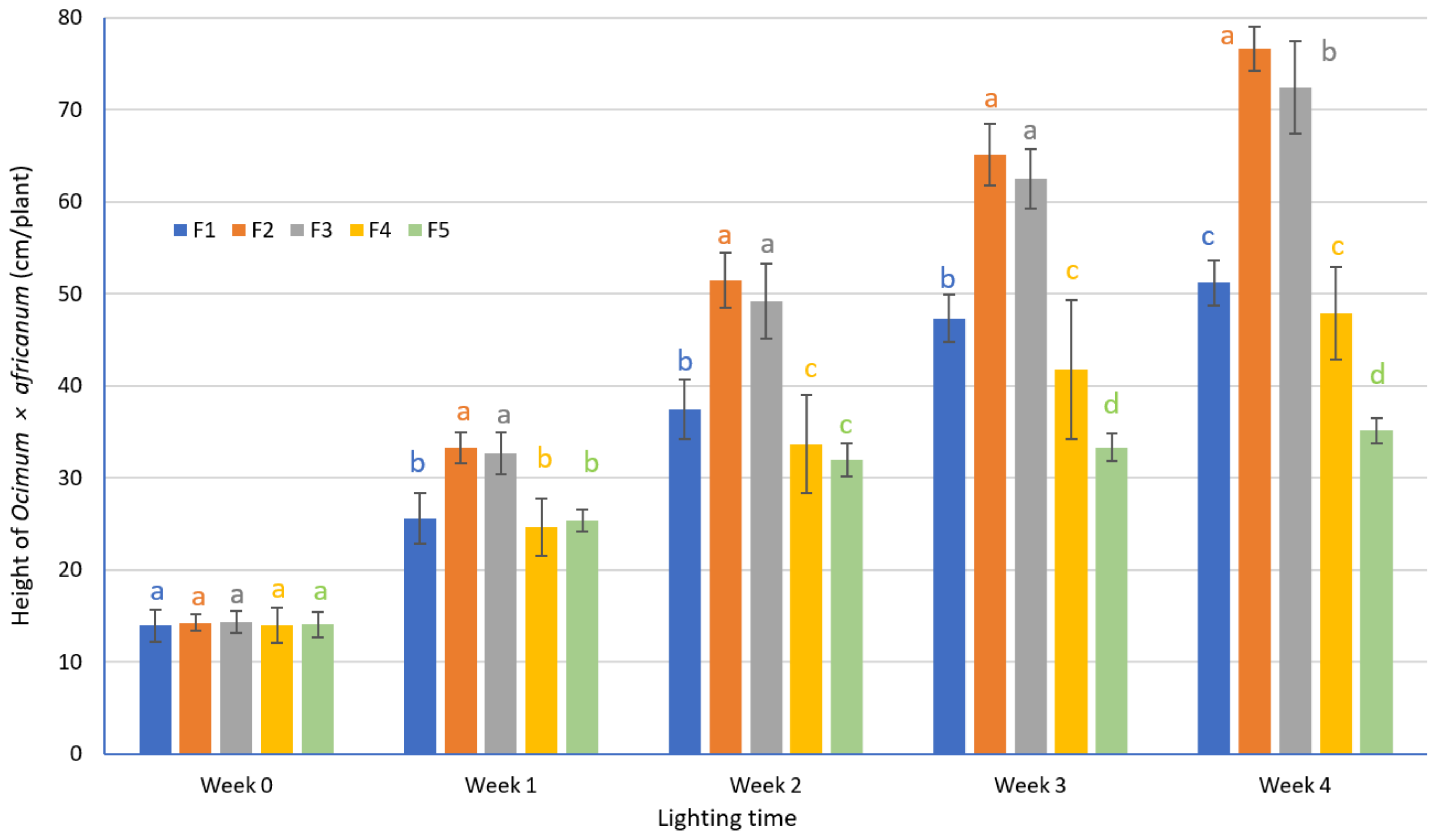
The height of *Ocimum* × *africanum* cultivated under different light conditions. (Note: Mean values followed by the same letter within data at each week are not statistically different for 0.05 significant level (*n* = 10). Statistical analyses were performed using IRRISTAT ver. 5.0 (International Rice Research Institute, Laguna, Philippines)).

The different light spectra had distinct effects on the growth, metabolite, and essential oil biosynthesis in the aerial parts of *Ocimum* × *africanum* Lour. (lemon basil). The plant height increased progressively throughout the four-week cultivation period under all five lighting treatments (F1–F5), although significant differences among treatments became apparent from week 1 onward. At the beginning of the experiment (week 0), plant height was similar among treatments, averaging approximately 14.11 cm. After one week, plants grown under F2 (UV-A:B:R:Fr = 6.60:45.15:29.23:19.02) and F3 (UV-A:B:R:Fr = 3.30:47.37:29.24:20.09) exhibited significantly greater heights (32.65–33.27 cm) than those under F1, F4, and F5 (24.63–25.58 cm). This trend became more pronounced during subsequent weeks. At week 2, plants exposed to F2 and F3 reached 51.45 and 49.18 cm, respectively, whereas those under F5 showed the lowest height (31.97 cm). By week 3, F2 produced the tallest plants (65.07 cm), followed closely by F3 (62.46 cm), while F5 remained significantly shorter (33.28 cm). At the end of the experiment (week 4), the maximum plant height was recorded under F2 (76.62 cm), which was statistically comparable to F3 (72.45 cm) but significantly higher (*p* < 0.01) than F1 (51.18 cm), F4 (47.84 cm), and F5 (35.11 cm). Overall, the F2 and F3 lighting conditions promoted superior stem elongation and plant height, whereas F5 resulted in the least plant height throughout the cultivation period (Figure 1).

Light conditions markedly affected biomass production and essential oil accumulation in lemon basil. Among the five treatments, F1 (B:G:R:Fr = 17.14:29.8:47.4:5.66) produced the highest fresh shoot yield (8.03 ± 0.77 ton·ha^−1^) and dry shoot yield (1.44 ± 0.14 ton·ha^−1^), which were significantly (*p* < 0.01) greater than those obtained under the other treatments. In contrast, plants grown under F5 exhibited the lowest biomass, with fresh and dry yields of only 1.61 ± 0.20 and 0.14 ± 0.02 ton·ha^−1^, respectively. Water content varied significantly among treatments and ranged from 82.14 ± 0.09% in F1 to 91.27 ± 0.12% in F5. Treatments F2 and F3 showed relatively high water contents (88.05–88.59%), whereas F4 exhibited an intermediate value (84.08%). The essential oil content of the shoots was strongly influenced by the lighting regime. The highest oil content was recorded in F4 (B:G:R:Fr = 13.85:43.50:39.30:3.35) (0.83 ± 0.00% *w*/*w*, dry basis), followed closely by F5 (0.82 ± 0.00%), whereas F2 and F3 produced significantly lower values (0.66 ± 0.00%). Although F4 exhibited the highest essential oil concentration, the greatest essential oil yield per hectare was obtained in F1 (10.89 ± 1.04 L·ha^−1^) due to its superior biomass production. Essential oil yield decreased progressively in F4 (8.58 ± 1.04 L·ha^−1^), F2 (5.59 ± 0.65 L·ha^−1^), and F3 (4.47 ± 0.91 L·ha^−1^), while F5 produced the lowest yield (1.16 ± 0.14 L·ha^−1^) (Table 1).

### 2.2. The Effect of LED Light Conditions on Essential Oil Composition of Ocimum × africanum

The chemical composition of the essential oils extracted from lemon basil varied moderately among the five lighting treatments, with a total of 12–15 compounds identified, representing 97.8–99.5% of the total oil. In all treatments, the oils were dominated by oxygenated monoterpenes, accounting for 70.6–91.4% of the total composition. The principal constituents were geranial (*trans*-citral) and neral, which together constituted the citral chemotype characteristic of lemon basil. Geranial was the most abundant compound in all samples, ranging from 41.1% in F5 to 49.9% in F3, while neral varied between 27.5% (F5) and 37.3% (F2). Consequently, the combined citral content (geranial + neral) exceeded 80% in treatments F1–F4 but decreased to approximately 68.6% under F5. Minor oxygenated monoterpenes, including linalool (0.8–2.3%), terpinen-4-ol (1.2–2.3%), nerol (trace–1.1%), and geraniol (trace–0.9%), were detected in all or most treatments. In contrast, sesquiterpene hydrocarbons increased markedly under F5, reaching 27.1%, compared with only 7.7–11.7% in F1–F4. This increase was mainly associated with elevated levels of (*E*)-*β*-caryophyllene (9.9%), germacrene D (6.1%), and (*E*)-*α*-bisabolene (5.3%), all of which were considerably higher than those observed under the other lighting conditions. Treatment F4 also showed a moderate enrichment of sesquiterpenes, with (*E*)-*β*-caryophyllene and germacrene D reaching 4.5% and 3.1%, respectively. Monoterpene hydrocarbons and benzene derivatives were detected only in trace amounts (<0.2%) in all treatments. Overall, the results indicate that while all lighting regimes maintained the citral-rich chemotype of lemon basil, treatment F5 induced a noticeable shift from oxygenated monoterpenes toward sesquiterpene hydrocarbons, thereby altering the relative proportions of secondary metabolites in the essential oil (Table 2).

**Figure 2:**
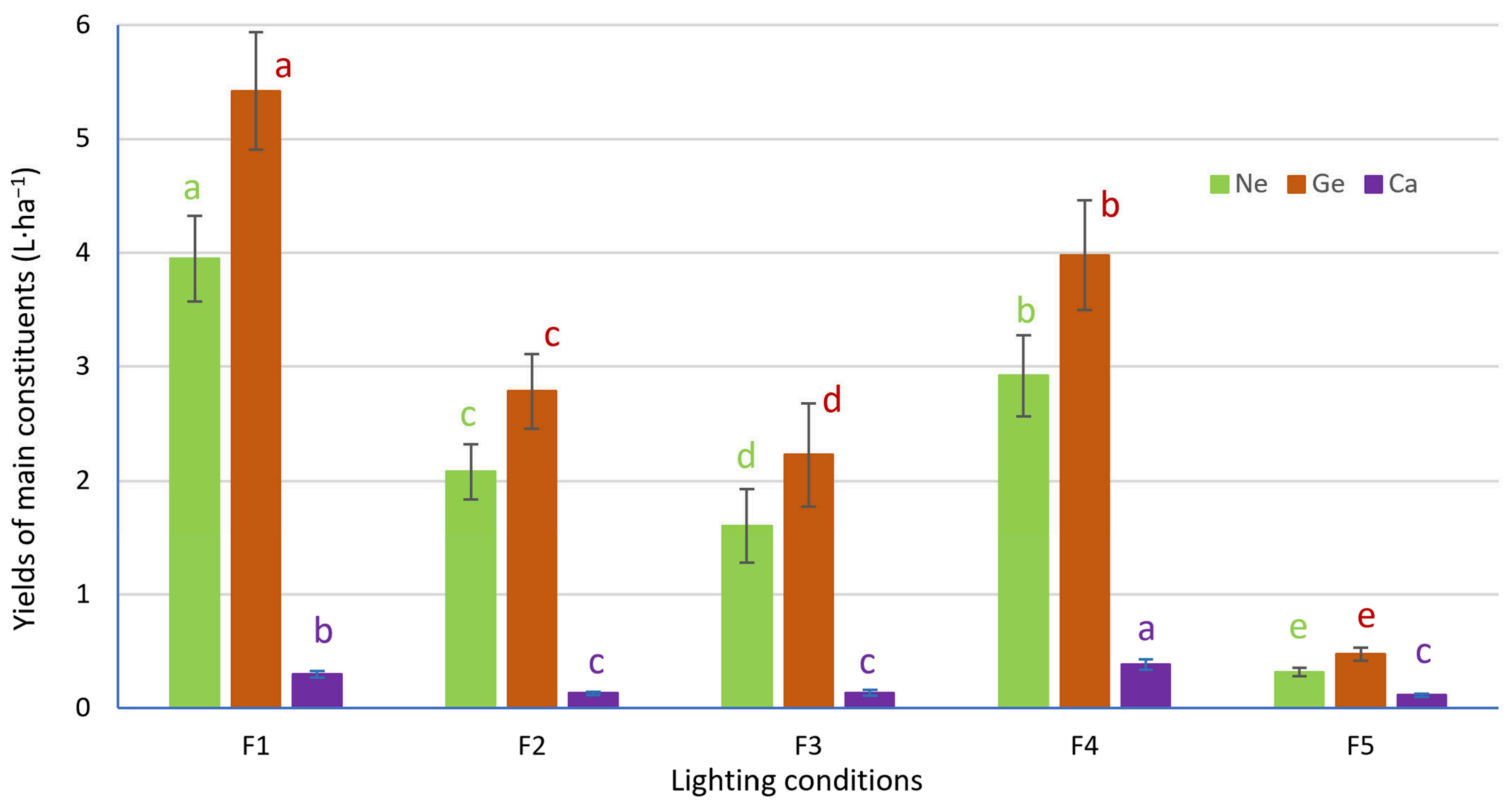
The calculated yields of main constituents in essential oils of *Ocimum* × *africanum* cultivated under different light conditions (note: Ne = neral, Ge = geranial = *trans*-citral, Ca = (*E*)-*β*-caryophyllene); mean values followed by the same letter within data of each constituent (Ne, Ge, and Ca) are not statistically different for 0.05 significant level (*n* = 10). Statistical analyses were performed using the software tool IRRISTAT ver. 5.0 (International Rice Research Institute, Laguna, Philippines).

The yields of the three main constituents, namely neral (Ne), geranial (Ge), and (*E*)-*β*-caryophyllene (Ca), in lemon basil essential oils varied significantly under five different lighting conditions. Among all treatments, F1 produced the highest yields of neral and geranial, reaching approximately 4.0 and 5.4 L·ha^−1^, respectively. F4 ranked second, with yields of about 2.9 L·ha^−1^ for neral and 4.0 L·ha^−1^ for geranial. In contrast, F5 resulted in the lowest yields for all constituents. The yield of (*E*)-*β*-caryophyllene was much lower than those of neral and geranial across all treatments, with the highest value observed under F4 (around 0.4 L·ha^−1^). Statistical analysis indicated significant differences among lighting treatments, as shown by different letters above the bars (*p* < 0.01) (Figure 2).

### 2.3. The Effect of LED Light Conditions on Pigments of Ocimum × africanum

**Figure 3:**
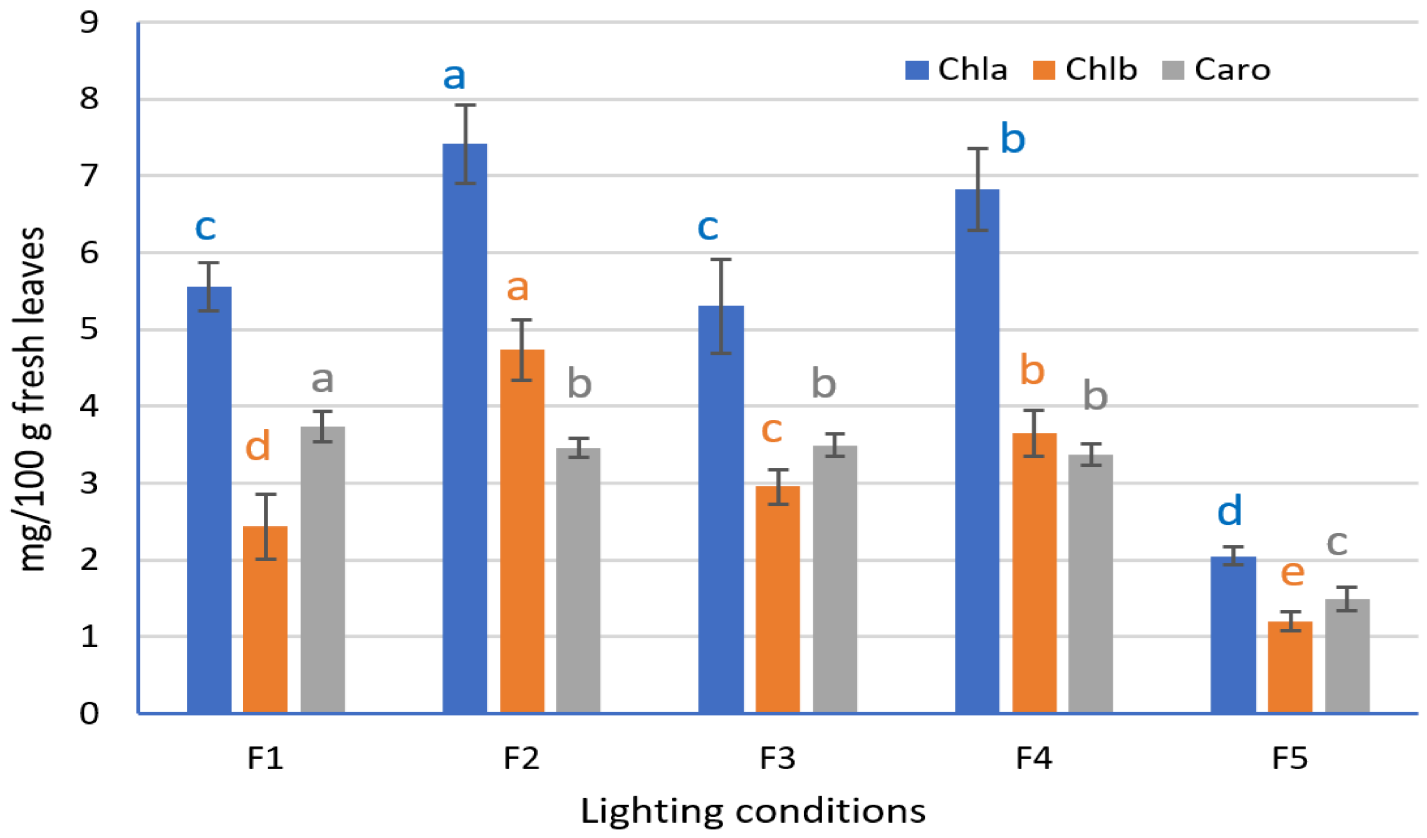
The concentrations of chlorophyll a (Chla), chlorophyll b (Chlb), and carotenoid (Caro) in fresh leaves of *Ocimum* × *africanum* cultivated under different light conditions; mean values followed by the same letter within data of each compound (Chla, Chlb, and Caro) are not statistically different for 0.05 significant level (*n* = 5). Statistical analyses were performed using the software tool IRRISTAT ver. 5.0 (International Rice Research Institute, Laguna, Philippines).

Chlorophyll a (Chla), chlorophyll b (Chlb), and carotenoids (Caro) are the major photosynthetic pigments involved in light harvesting and energy conversion in plants. The concentrations of these pigments in fresh leaves of lemon basil were significantly affected by different LED lighting conditions. Among the five treatments, F2 produced the highest contents of chlorophyll a and chlorophyll b, reaching 7.41 and 4.73 mg/100 g fresh leaves, respectively. These values were significantly greater (*p* < 0.01) than those observed under other treatments. F4 also supported substantial chlorophyll accumulation, whereas F1 and F3 exhibited intermediate levels. In contrast, F5 resulted in the lowest levels of both chlorophyll pigments. Carotenoid content followed a different trend, with the highest value recorded under F1 (3.74 mg/100 g fresh leaves), followed by F2, F3, and F4, while F5 again exhibited the lowest concentration. Overall, chlorophyll a levels were more abundant than chlorophyll b and carotenoid across all treatments (Figure 3).

**Figure 4:**
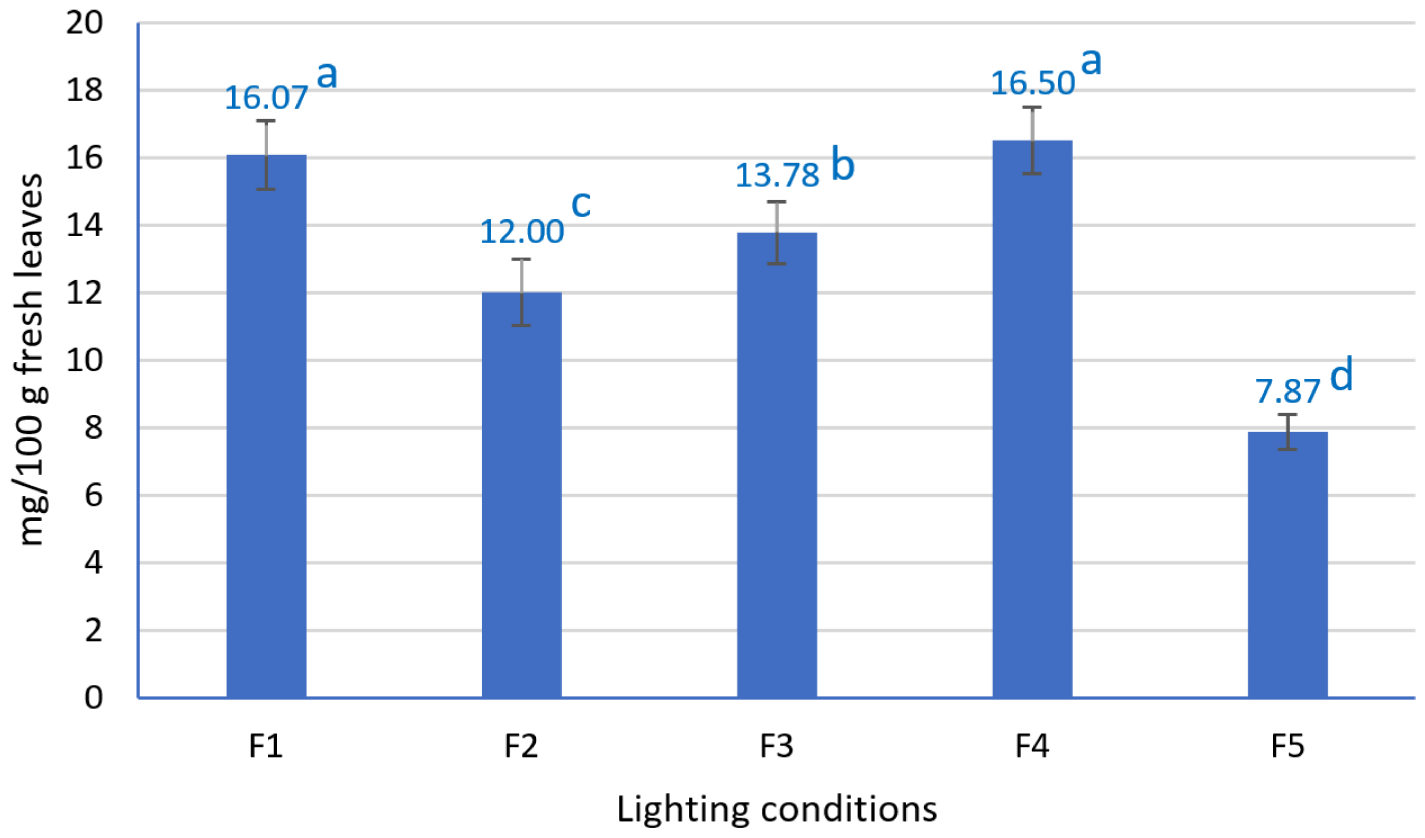
The concentrations of anthocyanin in fresh leaves of *Ocimum* × *africanum* cultivated under different light conditions; mean values followed by the same letter within the chart are not statistically different for 0.05 significant level (*n* = 5). Statistical analyses were performed using the software tool IRRISTAT ver. 5.0 (International Rice Research Institute, Laguna, Philippines)).

The anthocyanin concentration in fresh leaves of lemon basil was significantly influenced by different lighting conditions. The highest anthocyanin content was recorded under F4 (16.50 mg/100 g fresh leaves), followed closely by F1 (16.07 mg/100 g fresh leaves), and these two treatments were not significantly different (*p* > 0.05). Intermediate values were observed in F3 and F2, with concentrations of 13.78 and 12.00 mg/100 g fresh leaves, respectively. In contrast, F5 resulted in the lowest anthocyanin content, reaching only 7.87 mg/100 g fresh leaves (Figure 4).

### 2.4. The Effect of LED Light Conditions on Total Phenolic and Total Flavonoid of Ocimum × africanum

**Figure 5:**
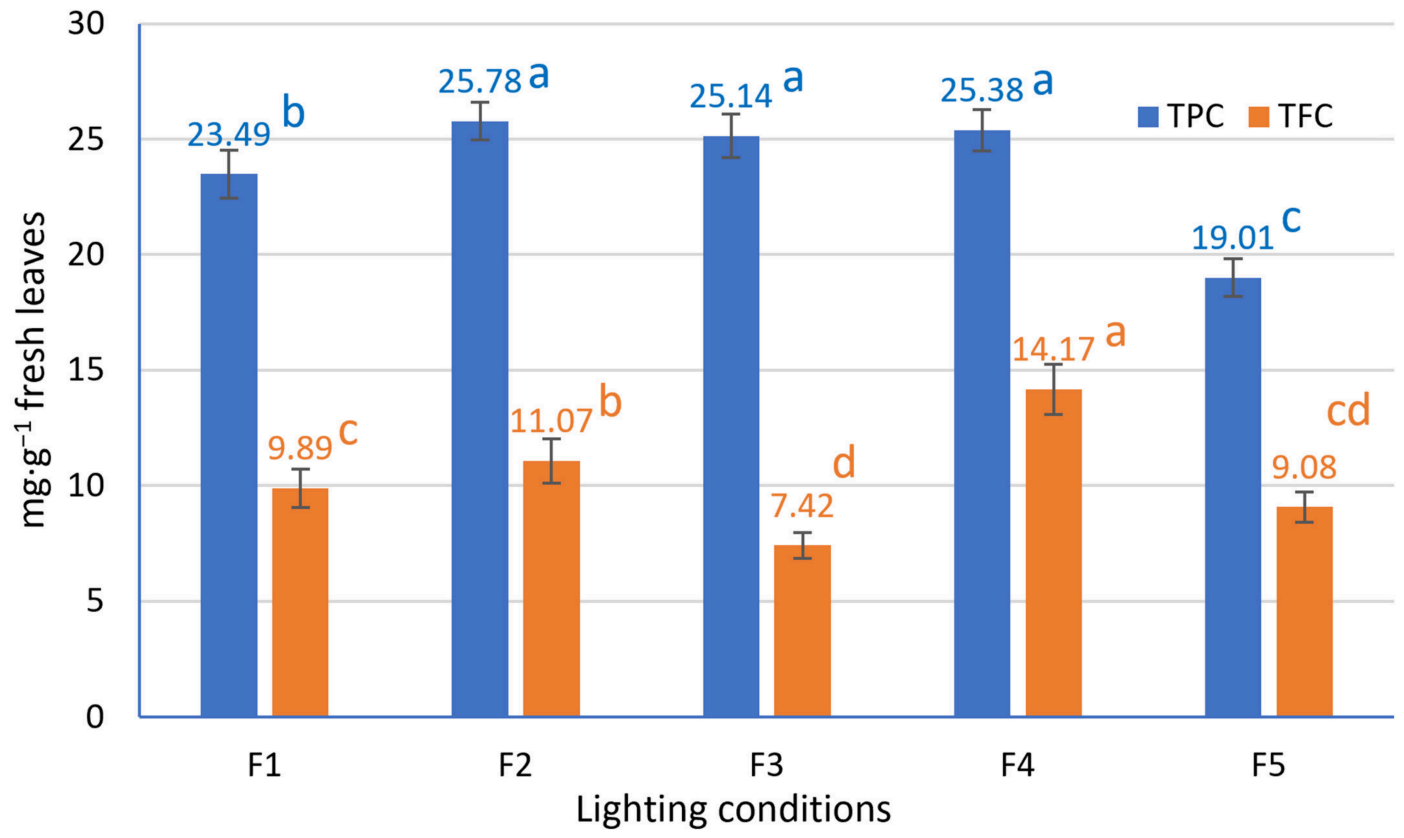
The concentrations of total phenolic content (TPC) and total flavonoid content (TFC) in fresh leaves of *Ocimum* × *africanum* cultivated under different light conditions; mean values followed by the same letter within the data of each compound are not statistically different for 0.05 significant level (*n* = 5). Statistical analyses were performed using the software tool IRRISTAT ver. 5.0 (International Rice Research Institute, Laguna, Philippines).

The total phenolic content (TPC) and total flavonoid content (TFC) in fresh leaves of lemon basil were significantly affected by different LED lighting conditions. The highest TPC values were observed under F2, F3, and F4, ranging from 25.14 to 25.78 mg·g^−1^ in fresh leaves, and these treatments were not significantly different from each other. In contrast, F5 showed the lowest TPC value (19.01 mg·g^−1^ in fresh leaves). For TFC, F4 produced the highest concentration (14.17 mg·g^−1^ in fresh leaves), followed by F2 (11.07 mg·g^−1^ in fresh leaves). The lowest TFC content was recorded under F3 (7.42 mg·g^−1^ in fresh leaves). Overall, F4 appeared to be the most favorable lighting condition for enhancing both phenolic and flavonoid accumulation (Figure 5).

## 3. Discussion

The relative increase in plant height of *Ocimum* × *africanum* varied markedly among the five lighting treatments throughout the cultivation period. All plants exhibited continuous growth over time; however, the magnitude of height increment differed considerably depending on the light condition. After one week, plants grown under F2 and F3 showed the greatest increases in height, reaching 133.31% and 128.00% above their initial values, respectively, whereas F4 and F5 exhibited the lowest increases (76.43% and 80.56%). This trend persisted during the subsequent weeks. By week 2, the height increase under F2 had reached 260.80%, followed by F3 (243.44%), while F5 showed the smallest increase (127.71%). At week 3, plants under F2 and F3 recorded height increments of 356.31% and 336.17%, respectively, which were substantially higher than those observed under F1 (238.85%), F4 (198.78%), and F5 (137.04%). At the end of the experiment (week 4), F2 produced the highest relative increase in plant height (437.31%), followed by F3 (405.94%). In contrast, plants exposed to F5 exhibited the lowest increase, reaching only 150.07% above the initial height. Overall, the F2 and F3 lighting regimes were the most effective in promoting stem elongation and plant growth, whereas F5 had the least stimulatory effect on height development. However, the height of lemon basil plants in the present study is higher than previously published values for many varieties with different species accessions [4].

As gene expression or enzyme activities were not measured, the mechanisms proposed in the present study remain speculative and require further investigation. The differences in plant height observed among the five lighting treatments can be attributed primarily to variations in spectral composition and daily light supply. Treatments F2 and F3 consistently produced the tallest plants. These two treatments were characterized by a high proportion of B light (45.15–47.37%) combined with relatively high Fr radiation (19.02–20.09%) under identical irradiance (220 ± 10 µmol·m^−2^·s^−1^) and photoperiod (16 h·d^−1^). Previous studies have demonstrated that Fr radiation plays an important role in regulating phytochrome-mediated shade-avoidance responses, promoting stem elongation and increasing plant height [34,45]. In addition, B light is essential for maintaining photosynthetic efficiency and supporting biomass accumulation through cryptochrome-mediated signaling pathways [46]. The high proportions of Fr and B spectral components in treatments F2 and F3 likely contributed to the most significant increases in plant height compared to the other treatments. Although F1 provided the highest proportion of R light (47.4%), its relatively low Fr fraction (5.66%) may have restricted shade-avoidance responses, resulting in less stem elongation than observed under F2 and F3. R light is highly efficient for photosynthesis because chlorophyll absorbs strongly in this spectral region; however, plant morphology is also strongly influenced by the R-to-Fr balance rather than R light alone [17]. Consequently, the limited Fr component in F1 may have reduced the stimulation of elongation growth. Plants grown under F4 exhibited lower height increments despite receiving the same PPFD and photoperiod as F1–F3. This treatment contained a relatively high proportion of G light (43.5%) and only a small fraction of Fr light (3.35%). Although G light can penetrate deeper into the canopy and contribute to photosynthesis, it is generally less effective than R and B light in regulating plant architecture and promoting shoot elongation [30]. The results obtained for the F1-F4 treatment in the present study are in agreement with previous findings demonstrating that a reduced R-to-Fr ratio stimulates stem elongation and enhances plant height, while an elevated R/Fr ratio restricts elongation growth, resulting in a more compact growth habit [47]. The lowest height increase was observed under F5, which reached only 150.07% above the initial height at week 4. Unlike the other treatments, F5 provided only a low DLI (2.35 mol·m^−2^·d^−1^) because the amount of sunlight and sky light transmitted through the greenhouse roof was only 25%. Light quantity is a major determinant of photosynthetic carbon assimilation and biomass production, and insufficient daily light exposure can severely limit plant growth [48]. Therefore, despite containing appreciable Fr radiation (17.26%), the low overall light input in F5 restricted plant development. This also highlights a limitation of the greenhouse control treatment, in which low irradiance may have masked the physiological effects of spectral composition. Overall, these results indicate that plant height in lemon basil is influenced by the interaction between spectral quality and light quantity. A lighting regime enriched with B and Fr lights under adequate irradiance and photoperiod conditions appears to be particularly effective in promoting stem elongation and vegetative height growth.

The marked differences in biomass production, water content, essential oil concentration, and essential oil yield among the five lighting treatments suggest that both light quality and daily light supply strongly influenced the physiological performance of lemon basil. Treatment F1 produced the highest fresh and dry biomass, whereas F5 resulted in the lowest productivity. F1 contained a balanced spectrum composed mainly of R light (47.4%), moderate G light (29.8%), and a smaller proportion of B light (17.14%), under a PPFD of 220 ± 10 µmol·m^−2^·s^−1^ with a 16 h photoperiod. R light is recognized as the most efficient light for photosynthesis because chlorophyll pigments absorb strongly in the R region, thereby enhancing carbon fixation and biomass accumulation [17]. In addition, G light penetrates deeper into leaf tissues and lower canopy layers, contributing to whole-canopy photosynthesis [30]. Consequently, although all four treatments (F1–F4) were maintained at an identical DLI of 12.67 mol·m^−2^·d^−1^, F1 resulted in greater dry matter accumulation and the highest biomass yield. This response is likely associated with the higher proportions of R and G light in F1, which may have synergistically enhanced photosynthetic efficiency, canopy light penetration, and carbon assimilation capacity compared with the other spectral treatments. In contrast, F5 received only a low supplemental light dose (2.35 mol·m^−2^·d^−1^), which limited photosynthetic carbon assimilation and consequently reduced biomass production. Similar observations were reported by Poorter et al. [48], who demonstrated that insufficient light availability is a major limiting factor for plant growth.

The water content of shoots was lowest in F1 (82.14%) and highest in F5 (91.27%). Because water content is inversely related to dry matter accumulation, plants producing greater amounts of structural carbohydrates generally exhibit lower moisture percentages. The higher water contents observed in F2, F3, and especially F5 indicate reduced accumulation of dry biomass relative to water uptake. Similar relationships between dry matter accumulation and tissue water content have been described in aromatic herbs grown under different light environments [49]. Interestingly, the highest essential oil concentration was observed in F4 (0.83%) and F5 (0.82%), despite their lower biomass compared with F1. F4 was characterized by the highest proportion of G light (43.5%) and a moderate R component, whereas F5 contained substantial Fr radiation together with UV-A and infrared (IR) light. Environmental stresses and suboptimal growth conditions frequently stimulate the biosynthesis of secondary metabolites, including terpenoids and essential oils, as part of plant defense mechanisms [50]. Several studies on *Ocimum* species showed that moderate stress or reduced growth rates may increase essential oil concentration even when biomass decreases [49,51]. Therefore, the elevated oil content in F4 and F5 may reflect a shift in carbon allocation from primary growth toward secondary metabolism. Nevertheless, essential oil yield per hectare depended on both oil concentration and biomass production. Although F4 exhibited the highest essential oil content, F1 generated the greatest essential oil yield (10.89 L·ha^−1^) because of its superior dry biomass production. Similar results have been reported in basil cultivation, where essential oil yield is determined primarily by the interaction between biomass accumulation and oil concentration rather than by oil content alone [52]. In contrast, the extremely low biomass in F5 limited total oil production, resulting in the lowest essential oil yield (1.16 L·ha^−1^) despite its relatively high oil concentration. Overall, these results indicate that different light spectra affect primary and secondary metabolism differently in lemon basil. While balanced R-dominant lighting with adequate a daily light integral favored biomass accumulation and maximized essential oil yield (F1), treatments associated with lower growth rates tended to enhance essential oil concentration. These results indicate that light quality not only affected plant growth and biomass accumulation but also influenced essential oil biosynthesis. The overall essential oil productivity of lemon basil depended on the combined effects of biomass production and oil concentration, with F1 providing the most favorable balance between these two factors. Thus, optimization of lighting strategies should consider the desired production objective, whether maximizing biomass, increasing essential oil concentration, or achieving the highest total oil yield.

The essential oil composition of lemon basil obtained in the present study was generally consistent with previous reports describing this species as a citral chemotype, characterized by the predominance of geranial and neral. Across all lighting treatments, geranial (41.1–49.9%) and neral (27.5–37.3%) were identified as the major constituents, together accounting for approximately 68.6–85.8% of the total oil composition. Similar findings were reported by Pisutthanan and Pisutthanan [5], who analyzed lemon basil populations collected from different regions of Thailand and found that neral (21.1–36.8%) and geranial (15.6–33.4%) were the principal components, confirming the existence of a citral-rich chemotype in this species. Likewise, Padalia et al. [53] reported total citral contents ranging from 55.0 to 75.5% in lemon basil essential oils and identified linalool, nerol, geraniol, and β-caryophyllene as important minor constituents.

Compared with previous studies, the present samples, particularly those from treatments F1–F4, exhibited relatively higher proportions of citral, indicating that these LED conditions effectively maintained the characteristic aroma profile of lemon basil. Minor oxygenated monoterpenes such as linalool (0.8–2.3%), terpinen-4-ol (1.2–2.3%), nerol, and geraniol were also detected, although their concentrations were lower than those reported in some earlier investigations. For example, Pisutthanan and Pisutthanan [5] observed linalool contents ranging from approximately 5 to 9% in certain populations, whereas the present study recorded values below 2.5%. In addition, methyl chavicol (estragole), which is frequently encountered in several *Ocimum* chemotypes, was detected only in trace amounts (<0.1%), indicating that the plants investigated here clearly belonged to the citral type rather than the estragole or linalool chemotypes commonly reported in basil species [54]. A remarkable difference was observed under treatment F5, where the proportion of oxygenated monoterpenes decreased to 70.6%, accompanied by a substantial increase in sesquiterpene hydrocarbons (27.1%). This shift was mainly associated with elevated levels of *(E)-β*-caryophyllene (9.9%), germacrene D (6.1%), and *(E)-α-*bisabolene (5.3%). Similar variations have been reported in previous studies, suggesting that environmental factors such as geographical origin, harvesting season, developmental stage, and cultivation conditions can significantly affect terpenoid biosynthesis in *Ocimum* species. Padalia et al. [53] demonstrated that harvest time and post-harvest processing markedly influenced the relative abundance of monoterpenes and sesquiterpenes in lemon basil. Likewise, Chang et al. [51] reported that different irradiance levels altered volatile oil composition in basil plants, indicating that light conditions can modulate secondary metabolism. Overall, despite the differences induced by the LED treatments, all samples retained the characteristic citral-rich profile of lemon basil. However, treatment F5 promoted a noticeable metabolic shift toward sesquiterpene accumulation, suggesting that light quality may influence the partitioning of carbon flux between monoterpene and sesquiterpene biosynthetic pathways. These findings highlight the potential of adjusting light environments to tailor the chemical profile of lemon basil essential oil according to specific industrial or pharmacological applications.

The significant differences in photosynthetic pigment accumulation observed under the five lighting treatments can be explained by the spectral composition of each LED combination and their effects on chlorophyll biosynthesis and photomorphogenic responses. Among the treatments, F2 (6.6% UV-A, 45.15% B, 29.23% R, and 19.02% Fr light) produced the highest chlorophyll a and chlorophyll b contents. The significantly higher chlorophyll content observed under F2 compared with F3 may be attributed to the synergistic interaction between UV-A and B radiation. Although both treatments contained a high proportion of B light, F2 provided twice the UV-A fraction of F3 (6.60% vs. 3.30%). UV-A and B lights are perceived primarily by cryptochromes and phototropins, which regulate chloroplast development, chlorophyll biosynthesis, and the expression of photosynthesis-related genes. Enhanced activation of these photoreceptors under F2 may have promoted chloroplast biogenesis and the accumulation of photosynthetic pigments, resulting in higher chlorophyll concentrations. Previous studies have demonstrated that B light enhances chlorophyll accumulation and photosynthetic capacity in many aromatic and medicinal plants [46,55]. It was shown that UV-A supplementation can increase chlorophyll content and photosynthetic capacity in horticultural crops by stimulating the development of the photosynthetic apparatus and enhancing light-harvesting efficiency [56]. Furthermore, the combined action of UV-A and B light was reported to exert a stronger regulatory effect on photomorphogenesis and chloroplast differentiation than B light alone through cryptochrome-mediated signaling pathways [57]. The presence of a moderate proportion of Fr radiation in F2 may also have improved photosystem balance and light-harvesting efficiency through the Emerson enhancement effect [58], contributing to the superior chlorophyll levels observed. F4, characterized by a high proportion of G light (43.5%) and moderate R light (39.3%), also maintained relatively high chlorophyll concentrations. G light can penetrate deeper into leaf tissues and lower canopy layers, thereby complementing B and R light and improving overall photosynthetic performance [30]. In contrast, F5 received only natural sunlight and sky light with a much lower daily light integral (2.35 mol·m^−2^·d^−1^), resulting in the lowest chlorophyll a and b contents. Insufficient light availability generally suppresses chlorophyll biosynthesis and reduces leaf photosynthetic activity [17]. Overall, the chlorophyll a and chlorophyll b contents in lemon basil of the present study are lower than previously published values, possibly due to the older age of the plants [40]. Unlike chlorophylls, carotenoid accumulation was highest under F1, which contained the greatest proportion of red light (47.4%), together with moderate B light and G light. Carotenoids function not only as accessory pigments in photosynthesis but also as photoprotective compounds that dissipate excess excitation energy and scavenge reactive oxygen species [59]. The relatively high R light fraction in F1 may have increased photosynthetic electron transport and induced greater demand for photoprotection, thereby stimulating carotenoid biosynthesis. Conversely, the lowest carotenoid content under F2 suggests that the enhanced chlorophyll production under B-enriched light reduced the requirement for carotenoid-mediated photoprotection. Overall, chlorophyll pigments were consistently more abundant than carotenoids in all treatments, reflecting their central role in light harvesting and carbon assimilation in lemon basil. The results confirm that the accumulation of chlorophylls and carotenoids in lemon basil is highly responsive to LED spectral composition and light intensity.

Anthocyanins are water-soluble flavonoid pigments responsible for the red, purple, and blue coloration observed in many plant tissues. Besides their role in pigmentation, anthocyanins function as powerful antioxidants and contribute to plant protection against various environmental stresses, including excessive light and oxidative damage. Their biosynthesis is highly responsive to external factors, particularly light quality and intensity, making anthocyanin accumulation an important indicator of plant physiological adaptation to different lighting conditions. The anthocyanin content of lemon basil leaves was significantly affected by the different LED lighting conditions, indicating that light quality strongly regulates flavonoid biosynthesis. The highest anthocyanin concentrations were observed under F4 (16.50 mg/100 g FW) and F1 (16.07 mg/100 g FW), whereas F5 produced the lowest value (7.87 mg/100 g FW). The elevated anthocyanin accumulation under F4 may be associated with its high proportion of G light (43.5%) combined with R light (39.3%), which could induce moderate photooxidative stress and stimulate the phenylpropanoid pathway. Anthocyanins are known to function as photoprotective pigments by absorbing excess radiation and scavenging reactive oxygen species generated under stressful light environments [60,61]. In contrast, the lower anthocyanin content observed under F2, despite exhibiting the highest chlorophyll *a* and chlorophyll *b* concentrations, suggests that B-enriched conditions favored photosynthetic pigment synthesis and efficient light utilization rather than the accumulation of protective secondary pigments. Similar inverse relationships between chlorophyll and anthocyanin accumulation have been reported in several species, where improved photosynthetic performance reduces the need for photoprotective anthocyanins [62]. Interestingly, F1 simultaneously promoted high carotenoid and anthocyanin contents, indicating enhanced photoprotection through both carotenoid-dependent quenching mechanisms and anthocyanin-mediated antioxidant activity. Carotenoids and anthocyanins often act synergistically to protect the photosynthetic apparatus against oxidative damage caused by excessive light [59]. Conversely, the low anthocyanin content under F5 corresponded with the lowest chlorophyll and carotenoid concentrations, which is likely attributable to the reduced daily light integral and limited photosynthetic activity under this treatment. Overall, the present results suggest that the accumulation of anthocyanins in lemon basil is closely linked to the balance between photosynthetic efficiency and photoprotective requirements imposed by different light environments.

TPC and TFC are important indicators of the antioxidant potential and phytochemical quality of plants. Phenolic compounds and flavonoids contribute significantly to plant defense mechanisms by scavenging reactive oxygen species and protecting tissues against environmental stresses. In addition to their physiological roles in plants, these secondary metabolites are associated with numerous health-promoting properties, including antioxidant, anti-inflammatory, antimicrobial, and anticancer activities. The accumulation of total phenolic compounds and flavonoids in lemon basil was markedly influenced by the spectral composition of the LED treatments, reflecting the close relationship between light quality, photosynthetic performance, and secondary metabolism. Treatments F2, F3, and F4 produced the highest TPC values (25.14–25.78 mg·g^−1^ FW), whereas F5 resulted in the lowest phenolic concentration. Similarly, F4 exhibited the greatest TFC (14.17 mg·g^−1^ FW), indicating that this lighting regime was particularly effective in stimulating flavonoid biosynthesis. Light is a major environmental factor regulating the phenylpropanoid pathway through the activation of enzymes such as phenylalanine ammonia-lyase (PAL), which plays a central role in the synthesis of phenolic compounds and flavonoids [17,61]. The relatively high proportions of B light in F2 and F3 and the high G light component in F4 may have enhanced the expression of genes involved in secondary metabolism, thereby promoting phenolic accumulation. Similar responses have been reported in basil and other medicinal plants, where blue-enriched LED spectra significantly increased phenolic and flavonoid contents [23,49]. Interestingly, the patterns of TPC and TFC showed clear relationships with the pigment data obtained in the present study. F2, which exhibited the highest chlorophyll *a* and chlorophyll *b* contents, also maintained high TPC values, suggesting that improved photosynthetic capacity provided sufficient carbon skeletons and reducing power for secondary metabolite biosynthesis. Enhanced photosynthesis has been shown to increase the availability of assimilates required for phenolic production [17]. In contrast, F4, which accumulated relatively high chlorophyll levels and showed the highest anthocyanin concentration, also produced the highest flavonoid content. Since anthocyanins belong to the flavonoid family, the simultaneous increase in anthocyanins and TFC under F4 indicates activation of the phenylpropanoid pathway and enhanced antioxidant defense mechanisms [60]. Moreover, F1, which exhibited the highest carotenoid concentration, showed only intermediate TPC and TFC values, suggesting that photoprotection under this treatment relied more heavily on carotenoid-mediated quenching rather than flavonoid accumulation. Carotenoids and phenolic compounds are known to complement each other in protecting plants against oxidative stress [59]. Conversely, F5 consistently produced the lowest chlorophyll, carotenoid, anthocyanin, and TPC values, which can be attributed to the low daily light integral and reduced photosynthetic activity under this treatment. Limited light availability decreases carbon assimilation and suppresses the biosynthesis of both photosynthetic pigments and secondary metabolites [48]. However, TOC and TFC values in lemon basil under all light treatments of the present study were higher than previously reported [38]. Overall, the present findings indicate that pigment accumulation and phenolic metabolism in lemon basil are closely interconnected, and that LED spectral composition can modulate both primary and secondary metabolism. Among the tested treatments, F4 provided the most favorable balance between pigment synthesis and antioxidant metabolite accumulation, thereby improving the phytochemical quality of the crop.

Data were analyzed using IBM SPSS Statistics version 27.0 (IBM Corp., Armonk, NY, USA). Prior to one-way ANOVA, the assumptions of normality and homogeneity of variances were evaluated using the Shapiro–Wilk and Levene’s tests, respectively. The results indicated that the majority of datasets satisfied these assumptions. Effect sizes (η^2^) were interpreted according to the conventional benchmarks proposed by Cohen [63], with values of 0.01, 0.06, and 0.14 indicating small, medium, and large effects, respectively, and were reported following current recommendations for statistical reporting [64]. In addition to statistical significance, effect size analysis showed that LED spectral treatments exerted large effects on most growth-related traits, including plant height, biomass accumulation, essential oil yield, and the yields of major essential oil constituents (η^2^ = 0.891–0.955). Likewise, large effects were observed for photosynthetic pigment-related parameters (η^2^ = 0.204–0.434). By contrast, the effects on TPC and TFC were of medium magnitude, with η^2^ values of 0.061 and 0.097, respectively.

**Figure 6:**
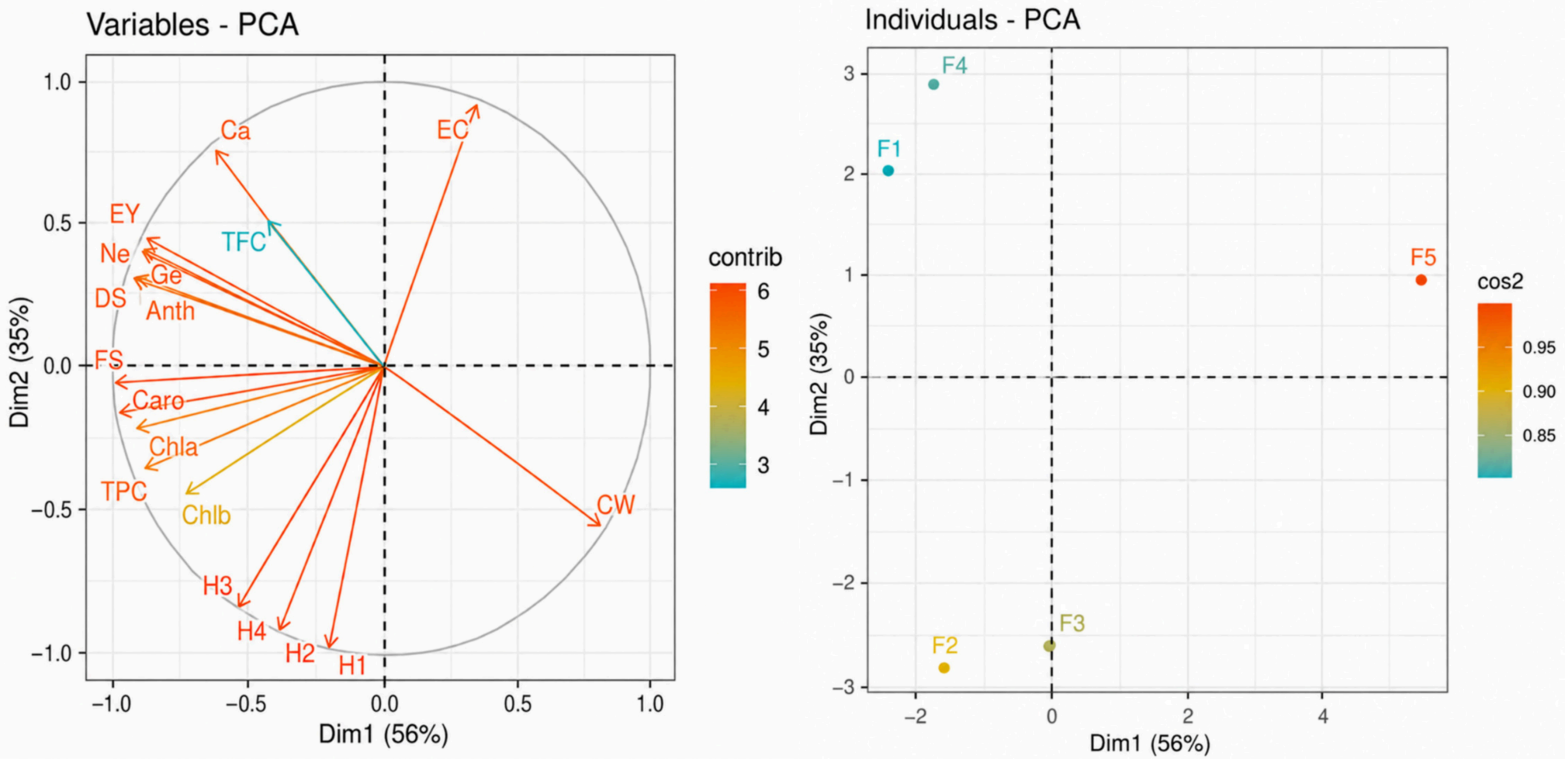
PCA of treatment effects and key contributors in *Ocimum* × *africanum*. Note: PCA analysis was performed using software CropGenoViz—Exploring Crop Genetic Diversity, ver. 1.0 (Nguyen, Trung Duc, https://ntducphd.shinyapps.io/cropgenoviz/; accessed on 2 July 2026); H1, H2, H3, and H4 = plant height after one, two, three and four weeks of lighting; FS = fresh yield of shoot; CW = water content of shoot; DS = dry yield of shoot; EC = essential oil content; EY = essential oil yield; Ne = neral; Ge = geranial; Ca = (*E*)-*β*-caryophyllene; Chla = chlorophyll a; Chlb = chlorophyll b; Caro = carotenoid; Anth = anthocyanin; TPC = total phenolic content; TFC = total flavonoid content.

Principal component analysis (PCA) was performed to provide an integrated overview of the relationships among growth, photosynthetic pigments, phenolic compounds, and essential oil traits under different lighting conditions (Figure 6). The first two principal components accounted for 91% of the total variance, with PC1 and PC2 explaining 56% and 35%, respectively, indicating that these two components adequately represented the overall variation among treatments. The PCA score plot (Individuals–PCA) showed a clear separation of the five lighting treatments, demonstrating that each light regime induced a distinct physiological and biochemical response in *Ocimum × africanum*. Notably, F1 and F4 clustered closely in the upper-left quadrant, suggesting similar overall responses, whereas F2 and F3 formed a separate cluster in the lower-left quadrant. In contrast, F5 was clearly separated from all LED treatments along the positive direction of PC1, indicating a markedly different response under the low-light control condition. The loading plot (Variables–PCA) further revealed that plant height, chlorophyll *a*, chlorophyll *b*, carotenoids, dry yield, and TPC were positively associated with one another, suggesting coordinated variation among plant growth, photosynthetic pigment accumulation, and phenolic metabolism. Likewise, anthocyanin, TFC, and the concentrations of neral, geranial, and (*E*)-*β*-caryophyllene were grouped in a similar direction, indicating positive correlations among these variables. Importantly, essential oil content was positioned in the upper-right quadrant of the loading plot and clearly separated from most other metabolites. This indicates that essential oil content is positively associated with PC2 but largely independent from the main cluster of vegetative growth, pigment accumulation, and phenolic traits. Its distinct orientation suggests that essential oil accumulation varied in a different pattern compared with other measured variables, and may represent a relatively independent response to light conditions. Conversely, water content was oriented in the opposite direction to most growth and biochemical traits, suggesting an inverse relationship with the accumulation of pigments and secondary metabolites across the lighting treatments. Overall, the PCA demonstrates that different light spectra generated distinct multivariate response patterns and highlights the close associations among plant growth, photosynthetic pigments, phenolic compounds, and essential oil composition in *Ocimum × africanum*.

Overall, the present study demonstrated that light environment plays a crucial role in modulating both primary and secondary metabolic processes in lemon basil (*Ocimum × africanum*). Differences in LED spectral composition significantly affected plant growth and productivity, as reflected by variations in plant height, biomass production, essential oil yield, and essential oil composition. In addition, light quality also strongly influenced the accumulation of photosynthetic pigments, phenolic compounds, and flavonoids, underscoring the potential of targeted spectral management as an effective approach for enhancing crop productivity and improving the phytochemical value of lemon basil. In future studies, it is recommended to include a standardized baseline treatment (e.g., R:B = 1:1 at the same PPFD) to more effectively isolate and evaluate the specific effects of additional spectral components such as G, UV-A, and Fr lights.

## 4. Materials and Methods

### 4.1. Plant Materials and Lighting Conditions

The experiment was conducted in growth chambers in the laboratory of the Technical University Darmstadt, Germany. The seeds of *Ocimum* × *africanum* (lemon basil), cultivar RADO 189 were purchased from the Rang Dong seed Ltd. company in Ho Chi Minh city, Vietnam. Lemon basil seeds were sown on 14 October 2025. After a 7-day germination period, seedlings were transplanted into small pots (110 mL volume; 7 cm diameter; 5.2 cm height). Sixteen days later, the plants were repotted into larger containers (750 mL volume; 12 cm diameter; 9.5 cm height). After an additional 5 days, the potted plants were transferred to five growth chambers with different lighting conditions for subsequent investigations. The techniques for planting, caring, fertilizing, and harvesting lemon basil plants were carried out according to a previous document [3]. Throughout the experiment, the ambient temperature was maintained at 22 ± 1 °C and the relative humidity at 68%. The lighting treatments were applied for 4 weeks, from 11 November to 9 December 2025. In four chambers from F1 to F4, a 16 h/8 h of light/dark photoperiod was used. The PPFD was set to 220 ± 10 µmol·m^−2^·s^−1^ and measured at canopy height using an MQ-650 ePAR meter with an underwater sensor (Apogee Instruments, Inc., Logan, UT, USA). The fifth chamber was a greenhouse receiving only natural global light (sunlight and sky light), with a transmission rate of 25%. Distinct spectral treatments were characterized using a CSS-45 Spectroradiometer of the company Gigahertz-Optik GmbH, Türkenfeld, Germany (Table 3).

To compare the spectral ratios between the five treatments, their common point is that they all contain R, B, and Fr spectra. The difference is that treatments F1 and F4 contain the highest amount of R and G, lowest amount of Fr, and no UV-A compared to the other three treatments. Treatments F2 and F3 have no G light, but contain the highest amount of B, relative high amount of Fr, and a small amount of UV-A. Treatment F5 contains the highest amount of Fr, and a small amount of UV-A and IR.

**Figure 7:**
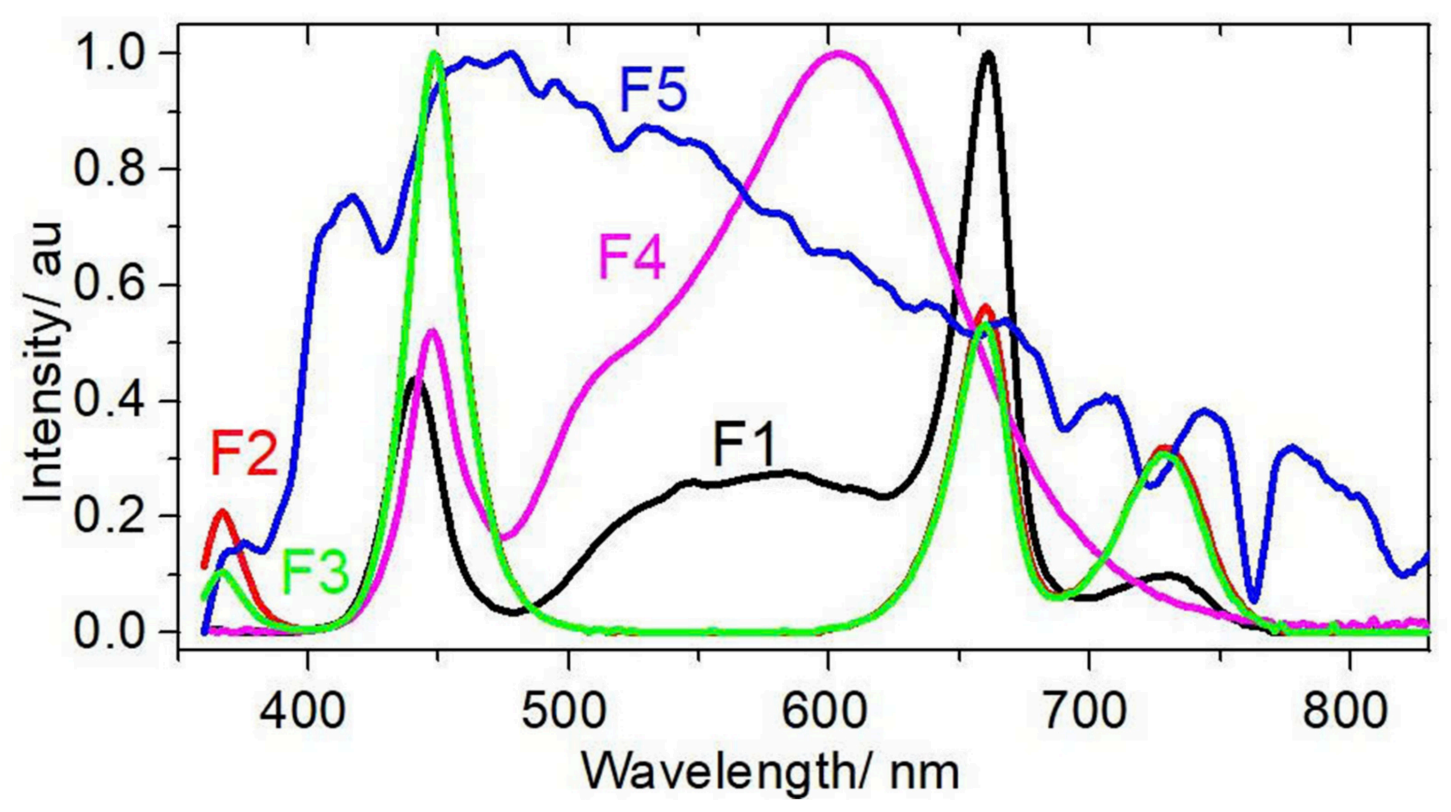
The relative spectra with different proportions of radiation regions.

Specifically, the four spectral formulations (F1–F4) were intentionally selected to represent distinct spectral environments rather than optimized lighting recipes. Their selection was guided by both the current knowledge of wavelength-specific physiological responses in plants and practical horticultural lighting applications. F1 and F4 were commercially available full-spectrum LED grow lights with the same spectral components but in different ratios: F1 (17.1% B, 29.8% G, 47.4% R, and 5.7% Fr) represents an R-enriched spectrum, whereas F4 (13.9% B, 43.5% G, 39.3% R, and 3.4% Fr) contains a G-rich spectrum. Comparing these two commercially relevant lighting systems allowed us to assess how differences in the balance among B, G, R, and Fr wavelengths influence plant growth and phytochemical accumulation under practical cultivation conditions. In contrast, F2 and F3 were custom-designed spectra developed to investigate the physiological roles of UV-A and Fr light more systematically. F2 (6.6 UV-A:45.15 B:29.23 R:19.02 Fr) was formulated as a B-enriched spectrum supplemented with relatively high proportions of UV-A and Fr, while F3 (3.3 UV-A:47.375 B:29.24 R:20.09 Fr) maintained nearly identical B, R, and Fr proportions but contained approximately half the UV-A fraction. This pairwise comparison enabled the specific contribution of UV-A intensity to be evaluated while minimizing changes in the remaining spectral components. F5 was positioned in the greenhouse as a low-light control (Figure 7).

**Figure 8:**
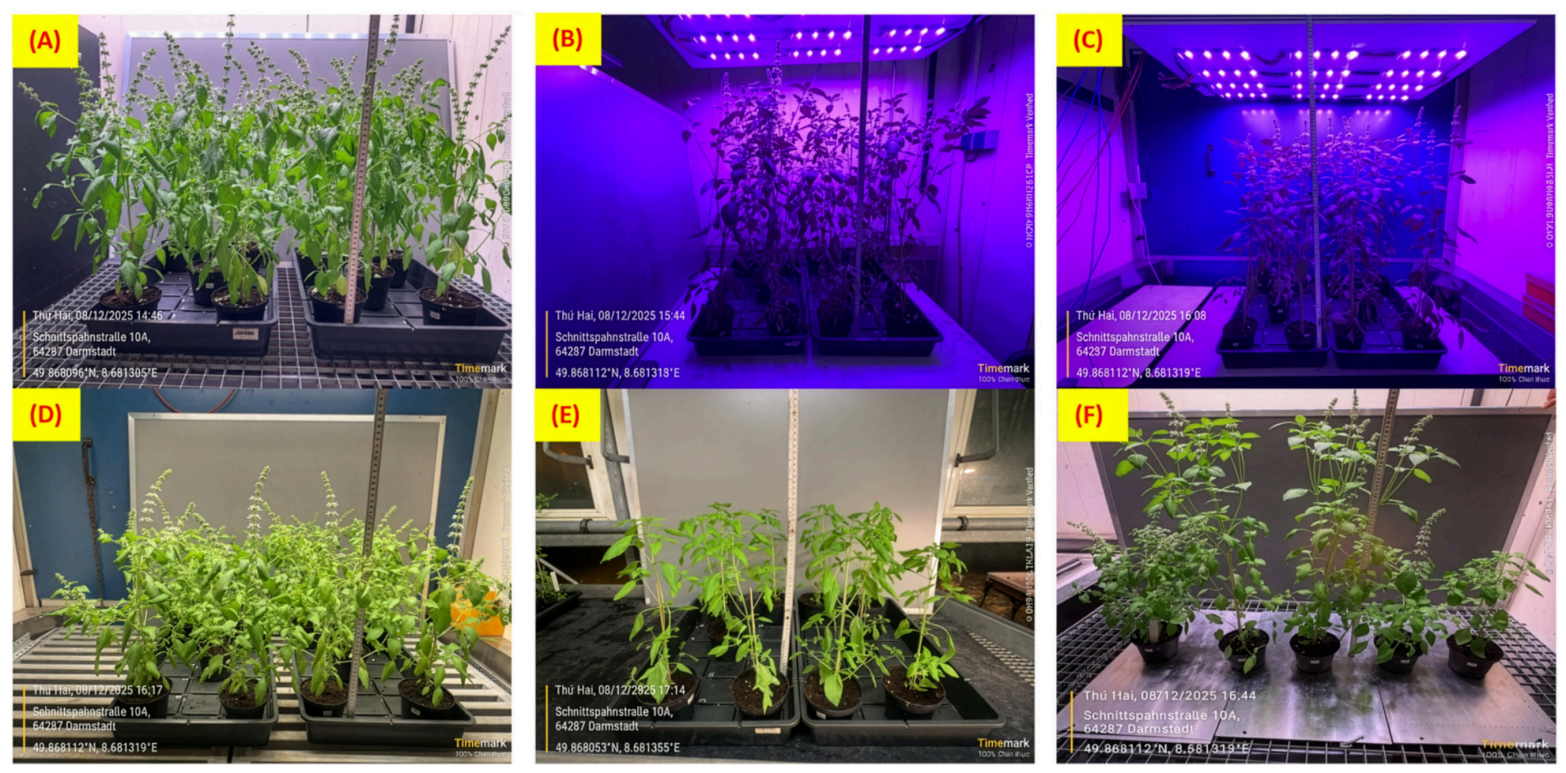
*Ocimum* × *africanum* plants cultivated in growth chambers under different light conditions. Note: the photos were taken one day before harvest; (**A**): F1 treatment, (**B**): F2 treatment, (**C**): F3 treatment, (**D**): F4 treatment, (**E**): F5 treatment, (**F**): comparing the morphology of the plants under 5 different lighting conditions, from the left to the right: F1, F2, F3, F4, and F5.

The LED lamps with a length of 1.2 m were installed above the growth chamber at an approximate distance of above 20 cm from the plant canopy to ensure uniform light distribution and spectral blending. The lamps were centrally positioned and aligned longitudinally along the growth chamber. Each treatment was conducted in one separated chamber and included 10 individual plants. The aerial parts of lemon basil were harvested at the end of the cultivation period, when most of the plants are full blooming, for subsequent analysis and evaluation. Each experimental treatment consisted of 10 plants grown under identical conditions (Figure 8). Plant height and biomass were determined using 10 plants per treatment, and the resulting values were used for statistical analysis. The values of plant physiological parameters were calculated per ha of growing, on a basis of density of 20 × 20 cm. For pigment and secondary metabolite analyses (Section 4.4, Section 4.5 and Section 4.6), five biological replicates were prepared for each treatment, with each replicate consisting of leaves pooled from two randomly selected plants. Sampling was standardized by selecting leaves from the fourth branch/node below the apical meristem. A total of 25 leaf samples were collected from lemon basil plants subjected to five different treatments. Immediately after harvest, the samples were wrapped in aluminum foil, flash-frozen in liquid nitrogen, and ground prior to subsequent biochemical analyses. Absorbance values were determined photometrically at appropriate wavelengths using a FoodALYT photometer (Omnilab-Laborzentrum GmbH and Co. KG, Bremen, Germany).

### 4.2. Essential Oil Isolation

Each lemon basil sample, consisting of 72–401 g of aerial biomass, was shredded and subjected to hydrodistillation for 2.5 h using a Clevenger-type apparatus [65]. The obtained essential oil was then separated and stored at −5 °C for subsequent analysis. For essential oil extraction, plant material from 10 plants within each treatment was divided into 2 portions (except for the sample from F5, which had only one part due to its small weight), and extraction was performed separately for each portion. Subsequently, the essential oils from each treatment were then pooled and analyzed in triplicate using GC-MS (each oil sample was injected three times) to determine its chemical composition.

### 4.3. Essential Oil GC-MS Analysis

GC-MS analysis was performed on a Thermo Scientific TRACE™ 1310 gas chromatograph coupled with an ISQ™ 7000 single quadrupole mass spectrometer (Thermo Scientific, Austin, TX, USA) operating in electron ionization (EI) mode at 70 eV. Separation was performed on an HP-5MS fused silica capillary column (60 m × 0.25 mm i.d., 0.25 μm film thickness). Helium was used as the carrier gas at a flow rate of 1.0 mL/min. The injector was set at 250 °C, with a 1 μL injection volume in split mode (1:100). The oven program started at 60 °C and was increased to 260 °C at 4 °C/min. Detector temperatures were maintained at 280 °C. For MS analysis, conditions included an interface temperature of 280 °C, electron ionization (EI) at 70 eV, a scan rate of 4.0 scans/s, and a mass range of 35–450 Da. Constituents were identified by comparing their relative retention indices (determined by co-injection with a homologous series of *n*-alkanes, C7–C30) and mass spectral fragmentation patterns with reference libraries (NIST2020, Wiley12, HPCH1607). Data was processed using Freestyle 1.8 and MassFinder 4.0. Relative concentrations were calculated from TIC peak areas without standardization. [43,44,66]. Additionally, the samples were analyzed with the same GC setup described above, but coupled with an Orbitrap Exploris (Thermo Scientific) operating in electron ionization (EI) mode at 70 eV in order to proof fragments with their exact masses.

### 4.4. Chlorophylls and Carotenoid Analysis

The contents of chlorophyll *a*, chlorophyll *b*, and carotenoid were determined using a spectrophotometric method based on the characteristic light absorption properties of photosynthetic pigments in organic solvents. Briefly, fresh leaf samples were ground in a porcelain mortar using liquid nitrogen. A sample amount of less than 300 mg was weighed into a 2 mL Eppendorf tube, and 1 mL of 90% methanol was added. The mixture was shaken on an orbital shaker for 30 min at room temperature, and then centrifuged for 30 min at 12,500 rpm using a filter funnel. Following extraction with 90% methanol, the pigments were dissolved in the extract and exhibited specific absorption maxima at characteristic wavelengths. Chlorophyll *a* showed maximum absorption at approximately 665 nm, and chlorophyll *b* at around 652 nm, whereas carotenoids absorbed predominantly at 470 nm. These photosynthetic pigments were extracted using 90% methanol, and 100 µL of the resulting extract was used for spectrophotometric analysis. Absorbance values were recorded photometrically at 665, 652, and 470 nm [67]. The contents of pigments were calculated according to the following equations:

Chlorophyll a (mg/L) = 16.82 × A(665) − 9.28 × A(652)(1)

Chlorophyll b (mg/L) = 36.92 × A(652) − 16.54 × A(665)(2)

Carotenoid (mg/L) = ([1000 × A470] − [1.91 × Chlorophyll a] − [95.15 × Chlorophyll b])/225(3)

### 4.5. Anthocyanin Analysis

Monomeric anthocyanins exhibit reversible structural transformations in response to pH changes, resulting in distinct color variations at pH 1.0 and pH 4.5. Under highly acidic conditions (pH 1.0), anthocyanins predominantly exist in the colored flavylium (oxonium) cation form, whereas at pH 4.5, they are mainly converted into the colorless hemiketal form. The leaf samples were processed similarly to the chlorophyll analysis method, with the exception that 80% methanol was used as the solvent and the samples were shaken overnight on an orbital shaker. Consequently, the difference in absorbance measured at 520 nm between these two pH conditions is directly proportional to the anthocyanin concentration. Anthocyanin content was expressed as cyanidin-3-glucoside equivalents using a molecular weight of 449.2 g·mol^−1^ and a molar extinction coefficient of 26,900 L mol^−1^·cm^−1^.

For the pH differential assay, two buffer solutions were prepared: 0.025 M potassium chloride buffer adjusted to pH 1.0 with HCl, and 0.4 M sodium acetate buffer adjusted to pH 4.5 with acetic acid. The pH values were verified using a pH meter (Hanna Instruments, Woonsocket, RI, USA). Equal proportions of extract and buffer were used, with 40 µL of sample extract mixed with 160 µL of the corresponding buffer solution in microplates. After an incubation period of 20–50 min, absorbance readings were recorded photometrically at 520 and 700 nm. Total monomeric anthocyanin content was determined according to the pH differential method described by Lee et al. [68], and calculated using the following equations:

A (absorbance) = (A_520_ − A_700_)_pH1_ − (A_520_ − A_700_)_pH4.5_ (4)

Anthocyanin content (mg/L) = (A × MW × DF × 1000)/(Ɛ × 1)(5)

where:

MW = molecular weight of cyanidin-3-O-glucoside (449.2 g/mol);

DF = Dilution factor (40:160 µL, adjustable if you do not see the pink color change);

Ɛ = molar extinction coefficient of cyanidin-3-O-glucoside (26,900 L/cm × mol);

l = path length of cuvette (typically = 1 cm).

Total anthocyanin content was calculated in the sample as mg per g of fresh weight (FW).

### 4.6. Total Phenolic and Total Flavonoid Concentration Analysis

To evaluate the effects of different light regimes on phenolic and flavonoid production, rapid spectrophotometric assays based on the chemical properties of these secondary metabolites were employed. Phenolic compounds are aromatic molecules containing free hydroxyl groups that become deprotonated under alkaline conditions, generating phenolate ions with reducing capacity. Upon addition of the Folin–Ciocalteu reagent, which contains phosphomolybdic and phosphotungstic acids, these phenolate ions reduce the metal complexes present in the reagent, leading to the formation of blue-colored molybdenum complexes. The intensity of the resulting blue coloration is proportional to the concentration of reducing phenolic compounds in the sample and was quantified photometrically at 735 nm. Gallic acid was used to make the standard curve at the concentrations of 250, 200, 150, 100 mg/L. A total of 50 independent calibration curves were generated during method validation and sample analysis.

Similarly, the determination of flavonoids relies on the reactivity of their hydroxyl groups. In the presence of aluminum chloride, these groups form stable chelate complexes with aluminum ions. Subsequent addition of sodium hydroxide promotes deprotonation of the hydroxyl groups, thereby enhancing complex formation and producing an intense red coloration. The absorbance of this colored complex was then measured photometrically at 510 nm to estimate flavonoid content. Catechin was used to make the standard curve at concentrations of 250, 200, 150, 100 mg/L. A total of 50 independent calibration curves were generated during method validation and sample analysis.

Based on these principles, total phenolic and flavonoid contents were determined following the methods described by Waterhouse [69], and Zhishen et al. [70], and Dou et al. [71], respectively, with appropriate modifications to the reagent and sample volumes.

### 4.7. Statistical Analysis

The effects of the lighting treatments on lemon basil were analyzed using a completely randomized design with one-way analysis of variance (ANOVA). Significant treatment effects were further evaluated by comparing means using the least significant difference (LSD) test at *p* ≤ 0.05. Statistical analyses were performed using IRRISTAT version 5.0 (International Rice Research Institute, Philippines). Prior to one-way ANOVA, the assumptions of normality and homogeneity of variances were evaluated using the Shapiro–Wilk and Levene’s tests, respectively. Eta-squared (η^2^) values were created to provide a quantitative measure of treatment effect size. These tests were performed using IBM SPSS Statistics version 27.0 (IBM Corp., Armonk, NY, USA). In addition, PAC analysis was performed using software CropGenoViz—Exploring Crop Genetic Diversity (Nguyen, Trung Duc, https://ntducphd.shinyapps.io/cropgenoviz/; accessed on 2 July 2026) to clarify overall trends in the dataset and highlight differences among treatments, as well as the main factors contributing to biomass, essential oil and secondary metabolites.

## 5. Conclusions

This study demonstrated that LED spectral composition markedly influenced growth, primary metabolism, and secondary metabolite accumulation in lemon basil (*Ocimum × africanum*). Different lighting regimes produced distinct responses: R-dominant lighting with moderate B and G, and low Fr promoted biomass production and carotenoid accumulation; B- and R-enriched lighting with moderate Fr and elevated UV-A enhanced stem elongation and chlorophyll accumulation; and a spectrum rich in R and G with moderate B, and limited Fr favored essential oil and other secondary metabolite accumulation. These findings highlight the importance of spectral quality in regulating lemon basil growth and phytochemical production. These findings raise several questions for future research, including whether UV-A proportions of up to 10% are suitable for sustained plant growth and whether future studies should prioritize spectral formulations optimized for biomass production or those enhancing essential oil accumulation. Further research should also integrate spectral optimization with energy consumption and economic assessments to develop precision-lighting strategies that are both biologically effective and economically viable for commercial controlled environment cultivation.

## Figures and Tables

**Table 1:** Biomass and essential oil yield of *Ocimum* × *africanum* cultivated under different light conditions.

Treatment	Fresh Yield of Shoot (ton·ha^−1^)	Water Content (%)	Dry Yield of Shoot (ton·ha^−1^)	Essential Oil Content (% *w*/*w*, Dry)	Essential Oil Yield (L·ha^−1^)
F1	8.03 ± 0.77 ^a^	82.14 ± 0.09 ^d^	1.44 ± 0.14 ^a^	0.76 ± 0.00 ^b^	10.89 ± 1.04 ^a^
F2	7.10 ± 0.83 ^b^	88.05 ± 0.10 ^b^	0.85 ± 0.10 ^c^	0.66 ± 0.00 ^c^	5.59 ± 0.65 ^c^
F3	5.90 ± 1.20 ^c^	88.59 ± 0.11 ^b^	0.67 ± 0.14 ^d^	0.66 ± 0.00 ^c^	4.47 ± 0.91 ^d^
F4	6.50 ± 0.79 ^bc^	84.08 ± 0.09 ^c^	1.04 ± 0.13 ^b^	0.83 ± 0.00 ^a^	8.58 ± 1.04 ^b^
F5	1.61 ± 0.20 ^d^	91.27 ± 0.12 ^a^	0.14 ± 0.02 ^e^	0.82 ± 0.00 ^a^	1.16 ± 0.14 ^e^

Note: Mean values followed by the same letter within a column are not statistically different for 0.05 significant level (*n* = 10). Statistical analyses were performed using the software tool IRRISTAT ver. 5.0 (International Rice Research Institute, Laguna, Philippines).

**Table 2:** Percentage-wise composition of essential oils of *Ocimum × africanum* cultivated under different light conditions (%).

Compounds ^a^	RI ^b^	RI ^c^	RI ^d^	F1 ^e^	F2 ^e^	F3 ^e^	F4 ^e^	F5 ^e^
*α*-Thujene	934	924	909–937	Tr	0.1	0.2	0.2	Tr
1,8-Cineole	1038	1026	1028–1038	Tr	Tr	Tr	0.1	Tr
Linalool	1110	1095	1091–1125	2.1	1.7	1.1	2.3	0.8
Terpinen-4-ol	1187	1174	1167–1191	1.4	2.3	2.2	2.1	1.2
Methyl chavicol (=Estragole)	1217	1195	1190–1206	Tr	Tr	Tr	Tr	0.1
Nerol	1238	1227	1202–1240	0.8	0.3	Tr	1.1	Tr
Neral	1244	1235	1236–1244	36.3	37.3	35.9	34.1	27.5
Geraniol	1262	1249	1243–1271	0.8	Tr	Tr	0.9	Tr
Geranial (=*trans*-Citral)	1273	1264	1269–1273	49.8	49.8	49.9	46.4	41.1
*α*-Copaene	1377	1374	1362–1387	0.4	0.3	0.4	0.5	1.2
(*E*)-*β*-Caryophyllene	1421	1417	1410–1442	2.8	2.4	3.1	4.5	9.9
*trans*-*α*-Bergamotene	1435	1432	1470	0.6	0.6	0.8	0.8	2.1
*α*-Humulene	1457	1452	1443–1462	0.4	0.5	0.6	0.6	1.8
Germacrene D	1483	1484	1470–1497	2.5	2.2	2.8	3.1	6.1
*δ*-Cadinene	1520	1522	1498–1539	0.2	0.1	0.2	0.2	0.7
(*E*)-*α*-Bisabolene	1543	NA	1544	1.4	1.6	2.0	2.0	5.3
Total				99.5	99.2	99.2	98.9	97.8
Monoterpene hydrocarbons				0.0	0.1	0.2	0.2	0.0
Oxygenated monoterpenes				91.2	91.4	89.1	87.0	70.6
Sesquiterpene hydrocarbons				8.3	7.7	9.9	11.7	27.1
Benzenoids				0.0	0.0	0.0	0.0	0.1
Number of compounds quantified				13	13	12	15	12

Note: ^a^ order of compounds eluted on the HP-5MS column; ^b^ RI: retention index of compounds on the HP-5MS column; ^c,d^ literature retention indices on HP-5MS column ^c^ [43] and ^d^ [44]; ^e^ each essential oil sample was analyzed in triplicate by GC-MS. The variation among technical replicate injections was negligible; therefore, standard deviations are not presented to improve table readability; NA: not available; Tr: trace (concentration < 0.1%).

**Table 3:** Light conditions in the cultivation of *Ocimum* × *africanum*.

Treatments	Spectral Distribution						Light Intensity (µmol·m^−2^·s^−1^)	Duration (h·d^−1^)	Daily Supplemental Light (mol·m^−2^·d^−1^)
	UV-A (360–400 nm) (%)	Blue (400–500 nm) (%)	Green (500–600 nm) (%)	Red (600–700 nm) (%)	Far Red (700–800 nm) (%)	IR (800–830 nm) (%)			
F1	0	17.14	29.8	47.4	5.66	0	220 ± 10	16	12.672
F2	6.6	45.15	0	29.23	19.02	0	220 ± 10	16	12.672
F3	3.3	47.37	0	29.24	20.09	0	220 ± 10	16	12.672
F4	0	13.85	43.5	39.3	3.35	0	220 ± 10	16	12.672
F5	4.56	25.43	26.5	21.75	17.26	4.5	0	0	2.35

## Data Availability

All data are available in this publication.

## Abbreviations

The following abbreviations are used in this manuscript:

B	blue
DLI	daily light integral
Fr	far red
G	green
IR	infrared
PCA	principal component analysis
PPFD	photosynthetic photon flux density
R	red
TFC	total flavonoid content
TPC	total phenolic content
Tr	trace
UV-A	ultraviolet-A
W	white

## References

[1] Zhang J.W., Li S.K., Wu W.J. (2009). The Main Chemical Composition and in Vitro Antifungal Activity of the Essential Oils of *Ocimum basilicum* Linn. Var. Pilosum (Willd.) Benth. Molecules.

[2] Vu X.P. (2000). Vietnamese Flora, Vol. 2: Mint Family—Lamiaceae Lindl. (Labiatae Juss.).

[3] La D.M., Luu D.C., Tran M.H., Nguyen T.T., Nguyen T.P., Tran H.T., Ninh K.B. (2001). Essential Oil Plant Resources in Vietnam.

[4] Vieira R.F., Simon J.E. (2006). Chemical Characterization of Basil (*Ocimum* spp.) Based on Volatile Oils. Flavour Fragr. J..

[5] Pisutthanan N., Pisutthanan S. (2009). Variability of Essential Oil Constituents of Ocimum Africanum. Asian Health Sci. Technol. Rep..

[6] Hamad A., Ramadhan M.B., Dewi D.Y.S., Djalil A.D., Hartanti D. (2020). Preservation Potential of Lemon Basil Essential Oil on Tofu: Development of a Natural Food Preservative. Proc. IOP Conf. Ser. Mater. Sci. Eng..

[7] Li X., Zhao Q., Chen H., Lai M., Zhao S., Feng W., Liu W., Li Y., Zhao M. (2025). Regional Discrimination of Volatile Organic Compounds in Ocimum× Citriodorum from Three Regions of China Using HS-SPME-GC-MS, HS-GC-IMS, E-Nose and E-Tongue. Ind. Crops Prod..

[8] Saleh S.S., El-Shayeb N.S.A. (2020). Impact of Spirulina Platensis and Stevia Rebaudiana on Growth and Essential Oil Production of Basil Ocimum Citriodorum. J. Agric. Rural Res..

[9] Al-Kateb H., Mottram D.S. (2014). The Relationship between Growth Stages and Aroma Composition of Lemon Basil Ocimum Citriodorum Vis. Food Chem..

[10] Pham T.H., Nguyen X.T., Nguyen P.N., Tran N.C.T., Thanh L.Q., Nguyen V.T., Nguyen T.H.T. (2022). Effect of Three Different Organic Fertilizers on Growth, Yield, and Essential Oil Content of Basil (*Ocimum basilicum* Var. Pilosum). J. Agric. Rural Dev..

[11] Paulus D., Valmorbida R., Ramos C.E.P. (2019). Productivity and Chemical Composition of the Essential Oil of Ocimum x Citriodorum Vis. According to Ontogenetic and Diurnal Variation. J. Appl. Res. Med. Aromat. Plants.

[12] Tahira R., Rehan T., Rehamn A., Naeemullah M. (2013). Variation in Bioactive Compounds in Different Plant Parts of Lemon Basil (*Ocimum basilicum* Var Citriodorum). Int. J. Innov. Sci. Math..

[13] Hamad A., Nuritasari A., Hartanti D. (2019). A Combination of Lemongrass and Lemon Basil Essential Oils Inhibited Bacterial Growth and Improved the Shelf Life of Chicken Fillets. J. Sci. Technol..

[14] Avetisyan A., Markosian A., Petrosyan M., Sahakyan N., Babayan A., Aloyan S., Trchounian A. (2017). Chemical Composition and Some Biological Activities of the Essential Oils from Basil Ocimum Different Cultivars. BMC Complement. Altern. Med..

[15] Jakovljević D., Topuzović M., Stanković M. (2019). Nutrient Limitation as a Tool for the Induction of Secondary Metabolites with Antioxidant Activity in Basil Cultivars. Ind. Crops Prod..

[16] Zabka M., Pavela R., Prokinova E. (2014). Antifungal Activity and Chemical Composition of Twenty Essential Oils against Significant Indoor and Outdoor Toxigenic and Aeroallergenic Fungi. Chemosphere.

[17] Taiz L., Zeiger E., Møller I.M., Murphy A. (2015). Plant Physiology and Development.

[18] Kume A., Akitsu T., Nasahara K.N. (2018). Why Is Chlorophyll b Only Used in Light-Harvesting Systems?. J. Plant Res..

[19] Eksi M., Rowe D.B. (2016). Green Roof Substrates: Effect of Recycled Crushed Porcelain and Foamed Glass on Plant Growth and Water Retention. Urban For. Urban Green..

[20] Livadariu O., Maximilian C., Rahmanifar B., Cornea C.P. (2023). LED Technology Applied to Plant Development for Promoting the Accumulation of Bioactive Compounds: A Review. Plants.

[21] Jakubczyk K., Szymczykowska K., Melkis K., Maciejewska-Markiewicz D., Nowak A., Muzykiewicz-Szymańska A., Skonieczna-Żydecka K. (2024). The Role of Light in Enhancing the Nutritional and Antioxidant Qualities of Basil, Mint and Lemon Balm. Foods.

[22] Wu W., Chen L., Liang R., Huang S., Li X., Huang B., Luo H., Zhang M., Wang X., Zhu H. (2025). The Role of Light in Regulating Plant Growth, Development and Sugar Metabolism: A Review. Front. Plant Sci..

[23] Dou H., Niu G., Gu M., Masabni J.G. (2017). Effects of Light Quality on Growth and Phytonutrient Accumulation of Herbs under Controlled Environments. Horticulturae.

[24] Kang S., Kim J.E., Zhen S., Kim J. (2022). Mild-Intensity UV-A Radiation Applied over a Long Duration Can Improve the Growth and Phenolic Contents of Sweet Basil. Front. Plant Sci..

[25] Somsong P., Weeplian T., Kiravittaya S., Tayjasanant T. (2024). Effects of Far-Red and UV-A Lighting on Phytochemicals and Antioxidant Activity in Late Growth Stage of Coriander (*Coriandrum sativum* L.). Proc. X Int. Symp. Light Hortic..

[26] Lee J.-H., Oh M.-M., Son K.-H. (2019). Short-Term Ultraviolet (UV)-A Light-Emitting Diode (LED) Radiation Improves Biomass and Bioactive Compounds of Kale. Front. Plant Sci..

[27] Chen Y., Li T., Yang Q., Zhang Y., Zou J., Bian Z., Wen X. (2019). UVA Radiation Is Beneficial for Yield and Quality of Indoor Cultivated Lettuce. Front. Plant Sci..

[28] Zhang Y., Kaiser E., Zhang Y., Zou J., Bian Z., Yang Q., Li T. (2020). UVA Radiation Promotes Tomato Growth through Morphological Adaptation Leading to Increased Light Interception. Environ. Exp. Bot..

[29] Terashima I., Fujita T., Inoue T., Chow W.S., Oguchi R. (2009). Green Light Drives Leaf Photosynthesis More Efficiently than Red Light in Strong White Light: Revisiting the Enigmatic Question of Why Leaves Are Green. Plant Cell Physiol..

[30] Smith H.L., McAusland L., Murchie E.H. (2017). Don’t Ignore the Green Light: Exploring Diverse Roles in Plant Processes. J. Exp. Bot..

[31] Zhen S., van Iersel M.W. (2017). Far-Red Light Is Needed for Efficient Photochemistry and Photosynthesis. J. Plant Physiol..

[32] Matysiak B., Kowalski A. (2021). The Growth, Photosynthetic Parameters and Nitrogen Status of Basil, Coriander and Oregano Grown under Different Led Light Spectra. Acta Sci. Pol. Hortorum Cultus.

[33] Nguyen D.T.P., Kitayama M., Lu N., Takagaki M. (2020). Improving Secondary Metabolite Accumulation, Mineral Content, and Growth of Coriander (*Coriandrum sativum* L.) by Regulating Light Quality in a Plant Factory. J. Hortic. Sci. Biotechnol..

[34] Park Y., Runkle E.S. (2017). Far-Red Radiation Promotes Growth of Seedlings by Increasing Leaf Expansion and Whole-Plant Net Assimilation. Environ. Exp. Bot..

[35] Zou J., Zhang Y., Zhang Y., Bian Z., Fanourakis D., Yang Q., Li T. (2019). Morphological and Physiological Properties of Indoor Cultivated Lettuce in Response to Additional Far-Red Light. Sci. Hortic..

[36] McAusland L., Lim M.T., Morris D.E., Smith-Herman H.L., Mohammed U., Hayes-Gill B.R., Crowe J.A., Fisk I.D., Murchie E.H. (2020). Growth Spectrum Complexity Dictates Aromatic Intensity in Coriander (*Coriandrum sativum* L.). Front. Plant Sci..

[37] Mat Daud Z., Awang Y., Ismail F., Mohamed M.T.M. (2017). Effects of Red and Blue Spectrum of Light Emitting Diodes (LEDs) on the Growth and Photosynthesis of Lemon Basil (*Ocimum* × *citriodorum*). Acta Horticulturae.

[38] Daud Z.M., Ismail M.F., Hakiman M. (2023). Effects of LED Red and Blue Spectra Irradiance Levels and Nutrient Solution EC on the Growth, Yield, and Phenolic Content of Lemon Basil (Ocimum Citriodurum Vis.). Horticulturae.

[39] Harakotr B., Srijunteuk S., Rithichai P., Tabunhan S. (2019). Effects of Light-Emitting Diode Light Irradiance Levels on Yield, Antioxidants and Antioxidant Capacities of Indigenous Vegetable Microgreens. Sci. Technol. Asia.

[40] Pitaloka N.D., Zahra A.M., Nugroho E., Simatupang H.K., Sinaga A.N.K., Annisa H.N., Rahmawati L. (2023). The Effect of Light-Emitting Diode, Planting Medium, and Nutrient Concentration on the Plant Growth and Chlorophyll Content of Lemon Basil. Proceedings of the 3rd International Conference on Smart and Innovative Agriculture (ICoSIA 2022).

[41] Garcia C., Lopez R.G., Currey C.J. (2025). Photosynthetic Daily Light Integral Has a Greater Impact on Basil Flowering than Photoperiod. HortScience.

[42] Shirokova A.V., Dzhatdoeva S.A., Ruzhitskiy A.O., Belopukhov S.L., Dmitrieva V.L., Luneva V.E., Dmitriev L.B., Kharchenko V.A., Kochkarov A.A., Sadykhov E.G. (2025). Treasures Induced by Narrow-Spectrum: Volatile Phenylpropanoid and Terpene Compounds in Leaves of Lemon Basil (*Ocimum*× *Citriodorum* Vis.), Sweet Basil (*O. basilicum* L.) and Bush Basil (*O. minimum* L.) under Artificial Light City Farm Conditions. Plants.

[43] Adams R.P. (2017). Identification of Essential Oil Components by Gas Chromatography, Mass Spectrometry.

[44] Linstrom P.J., Mallard W.G. (2025). NIST Chemistry Webbook, NIST Standard Reference Database Number 69.

[45] Franklin K.A., Whitelam G.C. (2005). Phytochromes and Shade-Avoidance Responses in Plants. Ann. Bot..

[46] Hogewoning S.W., Trouwborst G., Maljaars H., Poorter H., van Ieperen W., Harbinson J. (2010). Blue Light Dose–Responses of Leaf Photosynthesis, Morphology, and Chemical Composition of Cucumis Sativus Grown under Different Combinations of Red and Blue Light. J. Exp. Bot..

[47] Bantis F., Ouzounis T., Radoglou K. (2016). Artificial LED Lighting Enhances Growth Characteristics and Total Phenolic Content of Ocimum Basilicum, but Variably Affects Transplant Success. Sci. Hortic..

[48] Poorter H., Fiorani F., Pieruschka R., Wojciechowski T., van der Putten W.H., Kleyer M., Schurr U., Postma J. (2016). Pampered inside, Pestered Outside? Differences and Similarities between Plants Growing in Controlled Conditions and in the Field. New Phytol..

[49] Carvalho S.D., Schwieterman M.L., Abrahan C.E., Colquhoun T.A., Folta K.M. (2016). Light Quality Dependent Changes in Morphology, Antioxidant Capacity, and Volatile Production in Sweet Basil (*Ocimum basilicum*). Front. Plant Sci..

[50] Selmar D., Kleinwächter M. (2013). Stress Enhances the Synthesis of Secondary Plant Products: The Impact of Stress-Related over-Reduction on the Accumulation of Natural Products. Plant Cell Physiol..

[51] Chang X., Alderson P.G., Wright C.J. (2008). Solar Irradiance Level Alters the Growth of Basil (*Ocimum basilicum* L.) and Its Content of Volatile Oils. Environ. Exp. Bot..

[52] Simon J.E., Morales M.R., Phippen W.B., Vieira R.F., Hao Z. (1999). Basil: A Source of Aroma Compounds and a Popular Culinary and Ornamental Herb. Perspect. New Crops New Uses.

[53] Padalia R.C., Singh V.R., Bhatt G., Chauhan A., Upadhyay R.K., Verma R.S., Chanotiya C.S. (2018). Optimization of Harvesting and Post-Harvest Drying Methods of Ocimum Africanum Lour. for Production of Quality Essential Oil. J. Essent. Oil Res..

[54] Carović-Stanko K., Orlić S., Politeo O., Strikić F., Kolak I., Milos M., Satovic Z. (2010). Composition and Antibacterial Activities of Essential Oils of Seven Ocimum Taxa. Food Chem..

[55] Li Q., Kubota C. (2009). Effects of Supplemental Light Quality on Growth and Phytochemicals of Baby Leaf Lettuce. Environ. Exp. Bot..

[56] Verdaguer D., Jansen M.A.K., Llorens L., Morales L.O., Neugart S. (2017). UV-A Radiation Effects on Higher Plants: Exploring the Known Unknown. Plant Sci..

[57] Christie J.M., Blackwood L., Petersen J., Sullivan S. (2015). Plant Flavoprotein Photoreceptors. Plant Cell Physiol..

[58] Zhen S., Bugbee B. (2020). Far-red Photons Have Equivalent Efficiency to Traditional Photosynthetic Photons: Implications for Redefining Photosynthetically Active Radiation. Plant Cell Environ..

[59] Hashimoto H., Uragami C., Cogdell R.J. (2016). Carotenoids and Photosynthesis. Carotenoids in Nature.

[60] Gould K.S. (2004). Nature′ s Swiss Army Knife: The Diverse Protective Roles of Anthocyanins in Leaves. BioMed Res. Int..

[61] Zoratti L., Karppinen K., Luengo Escobar A., Häggman H., Jaakola L. (2014). Light-Controlled Flavonoid Biosynthesis in Fruits. Front. Plant Sci..

[62] Steyn W.J., Wand S.J.E., Holcroft D.M., Jacobs G. (2002). Anthocyanins in Vegetative Tissues: A Proposed Unified Function in Photoprotection. New Phytol..

[63] Cohen J. (1988). Statistical Power Analysis for the Behavioral Sciences.

[64] Lakens D. (2013). Calculating and Reporting Effect Sizes to Facilitate Cumulative Science: A Practical Primer for t-Tests and ANOVAs. Front. Psychol..

[65] Ministry of Health (2017). Vietnamese Pharmacopoeia.

[66] König W.A., Joulain D., Hochmuth D.H. (2004). Terpenoids and Related Constituents of Essential Oils.

[67] Lichtenthaler H.K., Buschmann C. (2001). Chlorophylls and Carotenoids: Measurement and Characterization by UV-VIS Spectroscopy. Curr. Protoc. Food Anal. Chem..

[68] Lee J., Durst R.W., Wrolstad R.E. (2005). (Collaborators) Determination of Total Monomeric Anthocyanin Pigment Content of Fruit Juices, Beverages, Natural Colorants, and Wines by the PH Differential Method: Collaborative Study. J. AOAC Int..

[69] Waterhouse A. (2003). Determination of Total Phenolics. Curr. Protoc. Food Anal. Chem..

[70] Zhishen J., Mengcheng T., Jianming W. (1999). The Determination of Flavonoid Contents in Mulberry and Their Scavenging Effects on Superoxide Radicals. Food Chem..

[71] Dou H., Niu G., Gu M., Masabni J.G. (2018). Responses of Sweet Basil to Different Daily Light Integrals in Photosynthesis, Morphology, Yield, and Nutritional Quality. HortScience.

